# Supplementary material for: Structure of the Nmd4-Upf1 complex supports conservation of the nonsense-mediated mRNA decay pathway between yeast and humans
Source: PLoS Biol. 2024 Sep 27;22(9):e3002821. doi: 10.1371/journal.pbio.3002821 (PMC11463774; doi:10.1371/journal.pbio.3002821)
Supplement: S1 Fig — (A) Superposition of the structure of Upf1-HD (color code indicated in the upper diagram and also used in panel B) bound to Nmd4 onto the structure of human UPF1-HD (light blue) bound to UPF2 (rmsd of 1.32 Å over 582 Cα atoms; PDB code: 2WJV; [53]). The largest difference between these structures is observed in the orientation of domain 1B. For the sake of clarity, yeast Nmd4, human UPF1-CH domain, and UPF2 region interacting with UPF1-CH have been omitted. (B) Superposition of the structure of Upf1-HD bound to Nmd4 onto the structure of human UPF1-HD (yellow) bound to ADP and phosphate (rmsd of 1.65 Å over 582 Cα atoms; PDB code: 2GK6; [52]). The largest difference between these structures is observed in the orientation of domain 1B. (C) Comparison of the crystal structures of Kluyveromyces lactis Nmd4 (blue; PDB code: 7QHY; [49]) and S. cerevisiae Nmd4 (as observed in our structure of the complex with Upf1-HD; the PIN domain and the arm are colored light and dark green, respectively; rmsd of 1.2 Å over 154 Cα atoms and 41% sequence identity). The amino acids matching with human SMG6 catalytic residues are shown as sticks. (D, E) Superposition of S. cerevisiae Nmd4 structure (as observed in our structure of the complex with Upf1-HD) onto human SMG5 (D; yellow; PDB code: 2HWY) or SMG6 (E; beige; PDB code: 2HWW) PIN domains (rmsd of 2 Å or 2.7 Å over 118 or 109 Cα atoms and 14% or 20% sequence identity, respectively; [34]). The amino acids matching with human SMG6 catalytic residues are shown as sticks. (F) Comparison of the endonuclease active site of human SMG6 PIN domain (wheat) with the corresponding region from S. cerevisiae Nmd4 (light green). The amino acids corresponding to human SMG6 catalytic residues are shown as sticks. (PDF) [file pbio.3002821.s001.pdf]

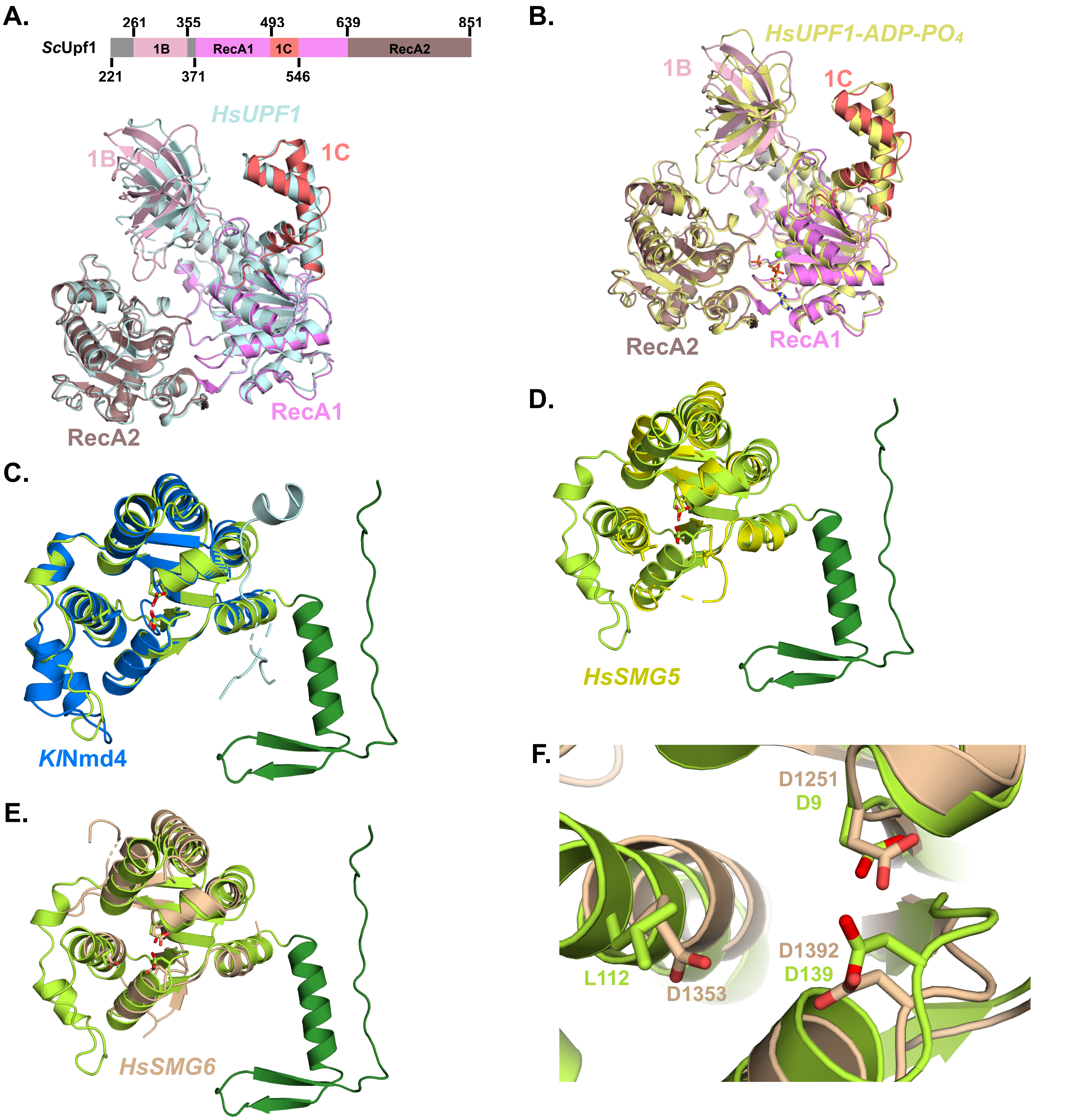

S1 Figure : Comparison of Upf1 and Nmd4 structures

A. Superposition of the structure of Upf1-HD (color code indicated in the upper diagram and also used in panel B) bound to Nmd4 onto the structure of human UPF1-HD (light blue) bound to UPF2 (rmsd of 1.32 Å over 582 Cα atoms; PDB code : 2WJV; [53]). The largest difference between these structures is observed in the orientation of domain 1B. For the sake of clarity, yeast Nmd4, human UPF1-CH domain and UPF2 region interacting with UPF1-CH have been omitted.

B. Superposition of the structure of Upf1-HD bound to Nmd4 onto the structure of human UPF1-HD (yellow) bound to ADP and phosphate (rmsd of 1.65 Å over 582 Cα atoms; PDB code : 2GK6; [52]). The largest difference between these structures is observed in the orientation of domain 1B.

C. Comparison of the crystal structures of *Kluyveromyces lactis* Nmd4 (blue; PDB code : 7QHY ; [49]) and *S. cerevisiae* Nmd4 (as observed in our structure of the complex with Upf1-HD; the PIN domain and the arm are colored light and dark green, respectively; rmsd of 1.2 Å over 154 Cα atoms and 41 % sequence identity). The amino acids matching with human SMG6 catalytic residues are shown as sticks.

D-E.Superposition of *S. cerevisiae* Nmd4 structure (as observed in our structure of the complex with Upf1-HD) onto human SMG5 (D; yellow; PDB code : 2HWY) or SMG6 (E; beige; PDB code : 2HWW) PIN domains (rmsd of 2 Å or 2.7 Å over 118 or 109 Cα atoms and 14 % or 20 % sequence identity, respectively; [34]). The amino acids matching with human SMG6 catalytic residues are shown as sticks.

F. Comparison of the endonuclease active site of human SMG6 PIN domain (wheat) with the corresponding region from *S. cerevisiae* Nmd4 (light green). The amino acids corresponding to human SMG6 catalytic residues are shown as sticks.
