# Supplementary material for: Structure of the Nmd4-Upf1 complex supports conservation of the nonsense-mediated mRNA decay pathway between yeast and humans
Source: PLoS Biol. 2024 Sep 27;22(9):e3002821. doi: 10.1371/journal.pbio.3002821 (PMC11463774; doi:10.1371/journal.pbio.3002821)
Supplement: S2 Fig — The panels are centered around Nmd4 Nmd4 W216 (A) or F178 (B) residues. The maps (contoured at 1σ) have been calculated using the Phenix.composite_omit_map program implemented in the phenix.refine program suite [77,78]. For the sake of clarity, the electron density map is only shown on some specific residues of the interface. (PDF) [file pbio.3002821.s002.pdf]

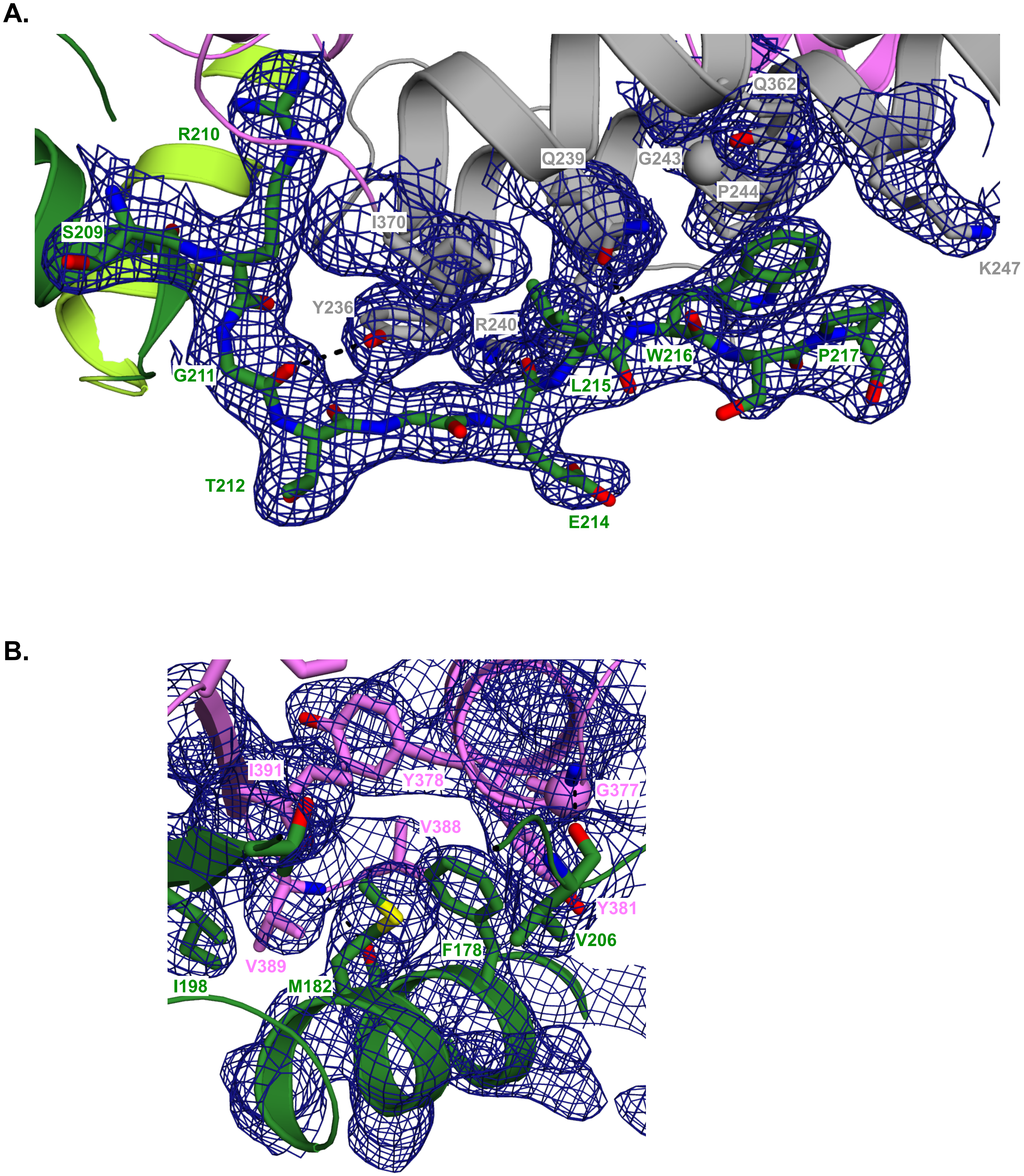

S2 Figure : Electron density 2mFo-DFc composite omit map illustrating the quality of the diffraction data. The panels are centered around Nmd4 Nmd4 W216 (A) or F178 (B) residues. The maps (contoured at 1σ) have been calculated using the Phenix.composite_omit_map program implemented in the phenix.refine program suite [77, 78]. For the sake of clarity, the electron density map is only shown on some specific residues of the interface.
