# Supplementary material for: Structure of the Nmd4-Upf1 complex supports conservation of the nonsense-mediated mRNA decay pathway between yeast and humans
Source: PLoS Biol. 2024 Sep 27;22(9):e3002821. doi: 10.1371/journal.pbio.3002821 (PMC11463774; doi:10.1371/journal.pbio.3002821)
Supplement: S3 Fig — Strictly conserved residues are in white on a black background. Partially conserved amino acids are shown in bold. Secondary-structure elements, as observed in the crystal structure of the S. cerevisiae Nmd4 protein bound to Upf1-HD, are shown above the alignment. Positions involved in the interface with Upf1-HD are indicated by black filled spheres below the alignment. Residues R210 and W216 mutated in this study are boxed in red. Domain boundaries are indicated above the alignment using the color code defined in Fig 1A. This figure was generated using the ENDscript server [76]. (PDF) [file pbio.3002821.s003.pdf]

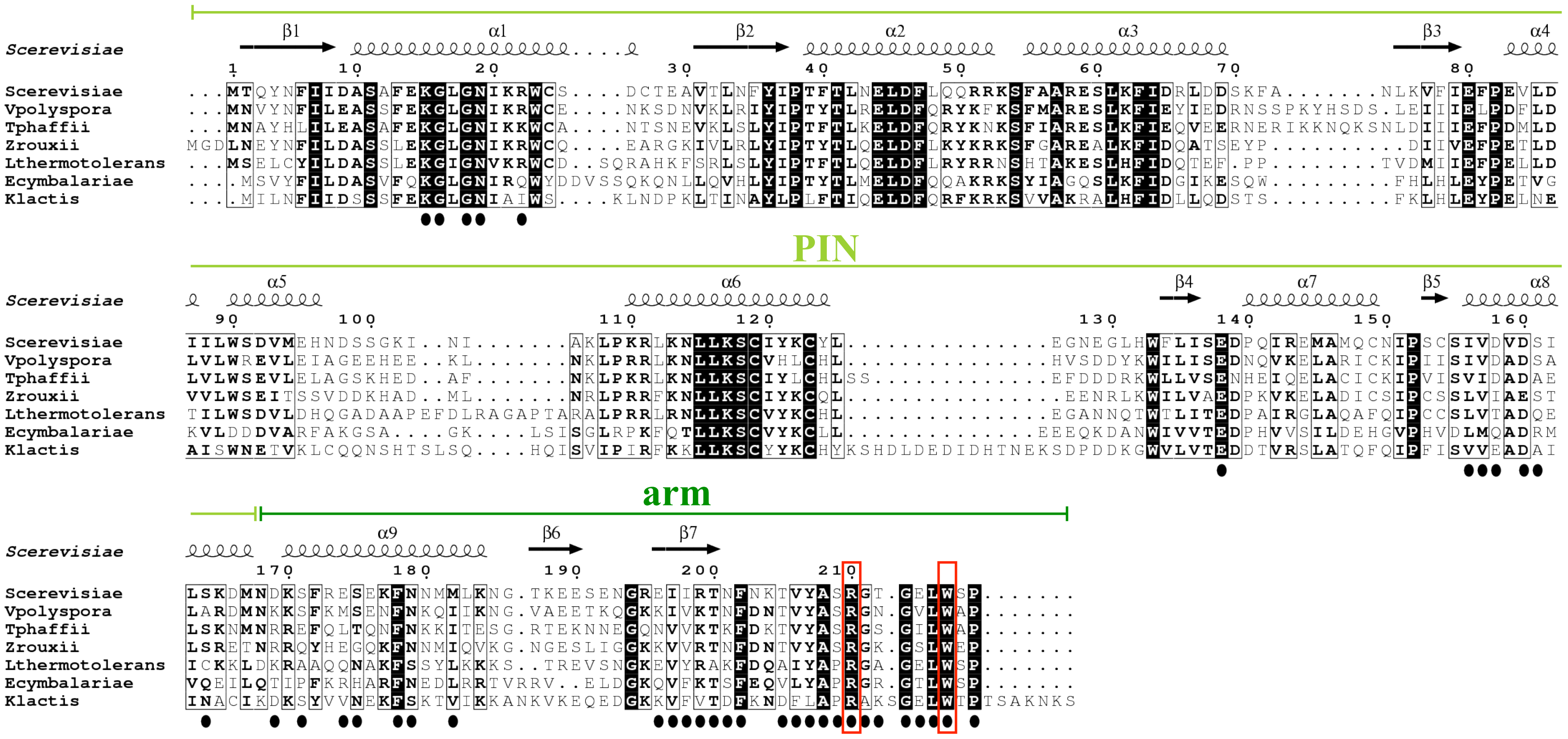

S3 Figure : Multiple sequence alignment of fungal Nmd4 orthologues.
Strictly conserved residues are in white on a black background. Partially conserved amino acids are shown in bold. Secondary-structure elements, as observed in the crystal structure of the *S. cerevisiae* Nmd4 protein bound to Upf1-HD, are shown above the alignment. Positions involved in the interface with Upf1-HD are indicated by black filled spheres below the alignment. Residues R210 and W216 mutated in this study are boxed in red. Domain boundaries are indicated above the alignment using the color code defined in Fig. 1A. This figure was generated using the ENDscript server (https://espript.ibcp.fr/ESPript/cgi-bin/ESPript.cgi; [76]).
