# Supplementary material for: Structure of the Nmd4-Upf1 complex supports conservation of the nonsense-mediated mRNA decay pathway between yeast and humans
Source: PLoS Biol. 2024 Sep 27;22(9):e3002821. doi: 10.1371/journal.pbio.3002821 (PMC11463774; doi:10.1371/journal.pbio.3002821)
Supplement: S5 Fig — (A) The Nmd4 « arm » domain is sufficient for the interaction with Upf1-HD in vitro. Pull-down experiments of Upf1-HD by different His-tagged Nmd4 domains. Input and elution (His) samples were analyzed on 12% SDS/PAGE and Coomassie blue staining. L: Molecular weight ladder. (B) The Nmd4 « arm » region is required for its interaction with Upf1 in vivo. Co-purification of Nmd4-FL-HA or Nmd4-PIN-HA with Upf1-FL-TAP was evaluated using an anti-HA immunoblot on total extracts and purified fractions. Strains expressing Nmd4-FL-HA and Nmd4-PIN-HA and native untagged Upf1 were used as negative controls. (PDF) [file pbio.3002821.s005.pdf]

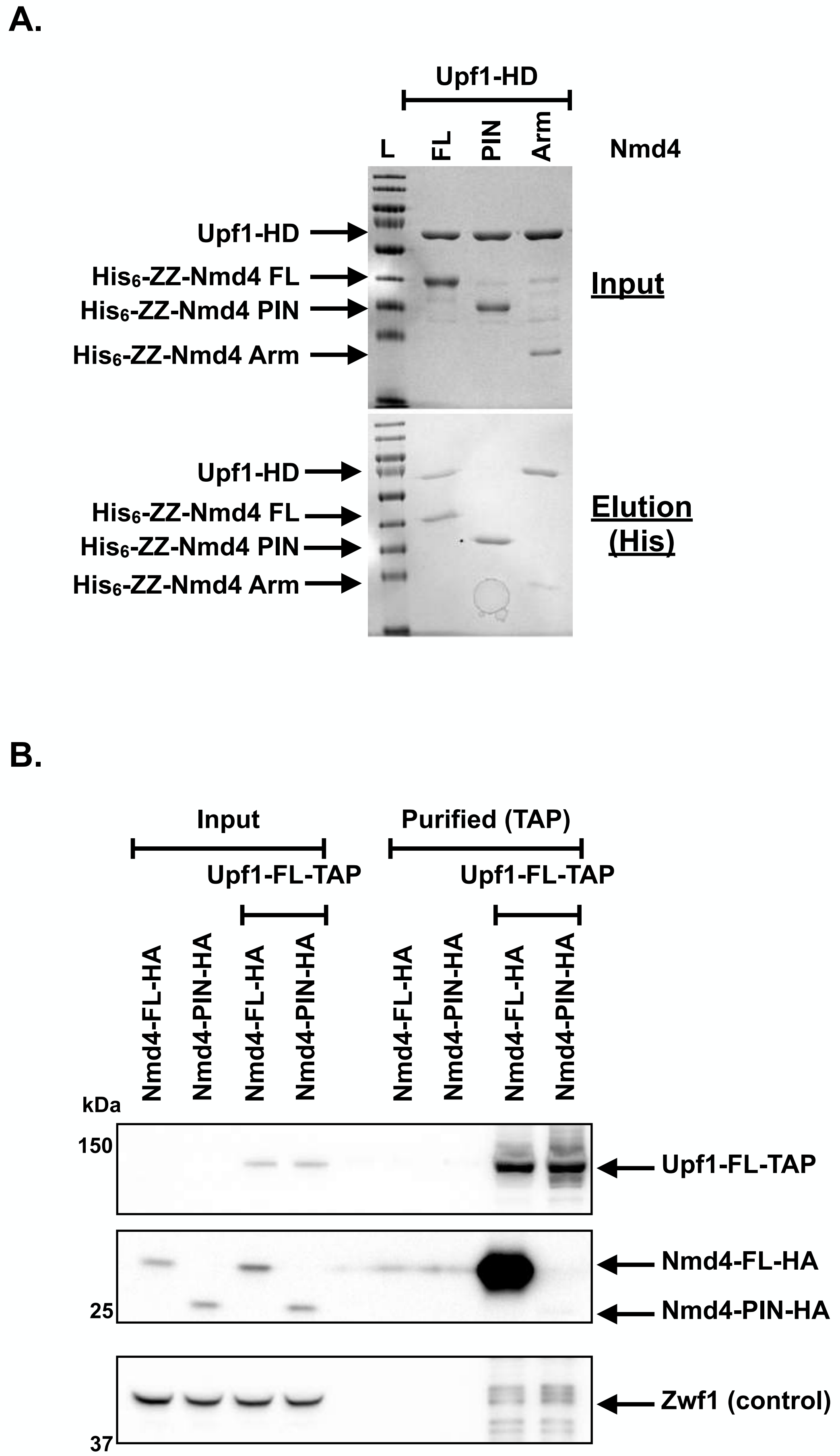

S5 Figure : The « arm » region from Nmd4 is sufficient for the interaction with Upf1-HD.

A. The Nmd4 « arm » domain is sufficient for the interaction with Upf1-HD *in vitro*. Pull-down experiments of Upf1-HD by different His-tagged Nmd4 domains. Input and elution (His) samples were analyzed on 12% SDS/PAGE and Coomassie Blue staining. L : Molecular weight ladder.

B. The Nmd4 « arm » region is required for its interaction with Upf1 *in vivo*. Co-purification of Nmd4-FL-HA or Nmd4-PIN-HA with Upf1-FL-TAP was evaluated using an anti-HA immunoblot on total extracts and purified fractions. Strains expressing Nmd4-FL-HA and Nmd4-PIN-HA and native untagged Upf1 were used as negative controls.
