# Supplementary material for: Structure of the Nmd4-Upf1 complex supports conservation of the nonsense-mediated mRNA decay pathway between yeast and humans
Source: PLoS Biol. 2024 Sep 27;22(9):e3002821. doi: 10.1371/journal.pbio.3002821 (PMC11463774; doi:10.1371/journal.pbio.3002821)
Supplement: S6 Fig — Interaction of Upf1-HD, His6-ZZ-Nmd4-FL, His6-ZZ-Nmd4-FL/Upf1-HD complex, and His6-ZZ with RNA poly(U)30 studied by ITC. The data underlying this figure can be found in S6 Data. (PDF) [file pbio.3002821.s006.pdf]

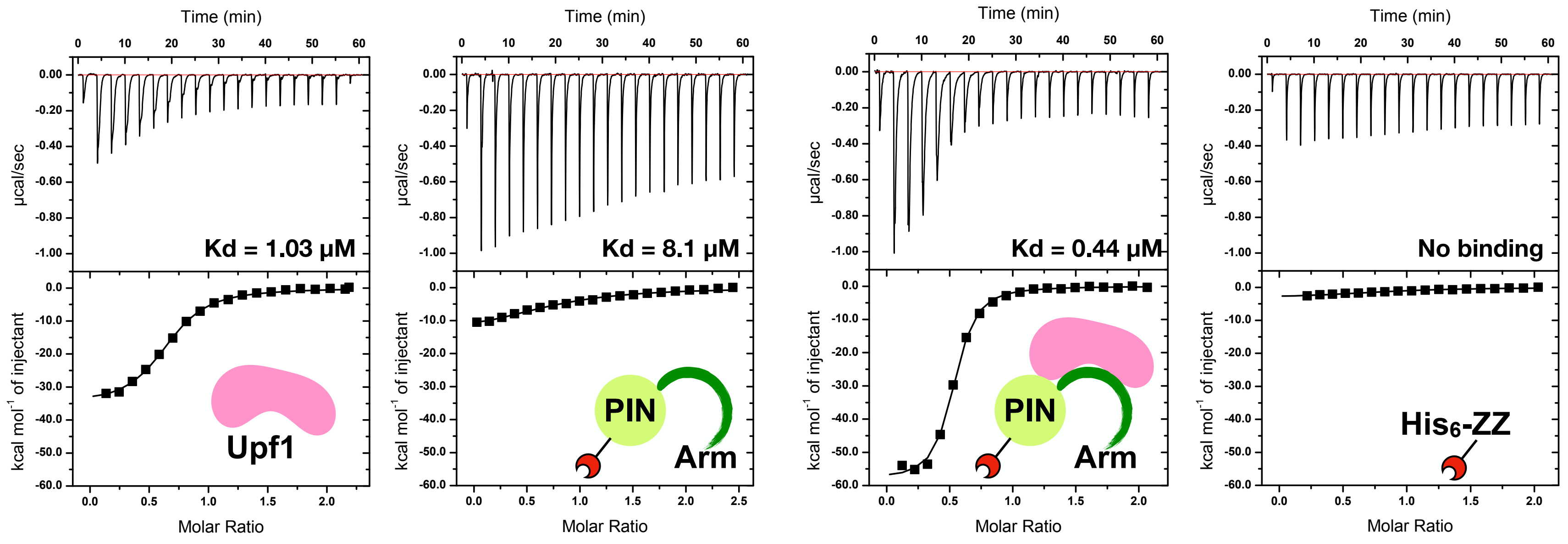

S6 Figure : Effect of Nmd4 on Upf1-HD RNA binding activity.
Interaction of Upf1-HD, His_6-ZZ-Nmd4-FL, His_6-ZZ-Nmd4-FL/Upf1-HD complex and His_6-ZZ with RNA poly$(U)_{30}$ studied by ITC. The data underlying this figure can be found in S6 Data.

S6 Figure
