# Supplementary material for: Structure of the Nmd4-Upf1 complex supports conservation of the nonsense-mediated mRNA decay pathway between yeast and humans
Source: PLoS Biol. 2024 Sep 27;22(9):e3002821. doi: 10.1371/journal.pbio.3002821 (PMC11463774; doi:10.1371/journal.pbio.3002821)
Supplement: S7 Fig — (A) Size exclusion chromatography (S200 16/60) profiles obtained for pure Upf1-HD WT and mutant proteins, showing very similar retention times for these different proteins. The Coomassie stained SDS-PAGE analysis of the proteins present in the main peak is shown for every protein (WT and mutants) to show the purity of the final proteins. MW: Molecular weight ladder. (B) Size exclusion chromatography (S75 16/60) profiles obtained for pure His6-ZZ-Nmd4 WT and mutant proteins, showing very similar retention times for these different proteins. The Coomassie stained SDS-PAGE analysis of the proteins present in the main peak is shown for every protein (WT and mutants) to show the purity of the final proteins. MW: Molecular weight ladder. The data underlying this figure can be found in S7 Data. (PDF) [file pbio.3002821.s007.pdf]

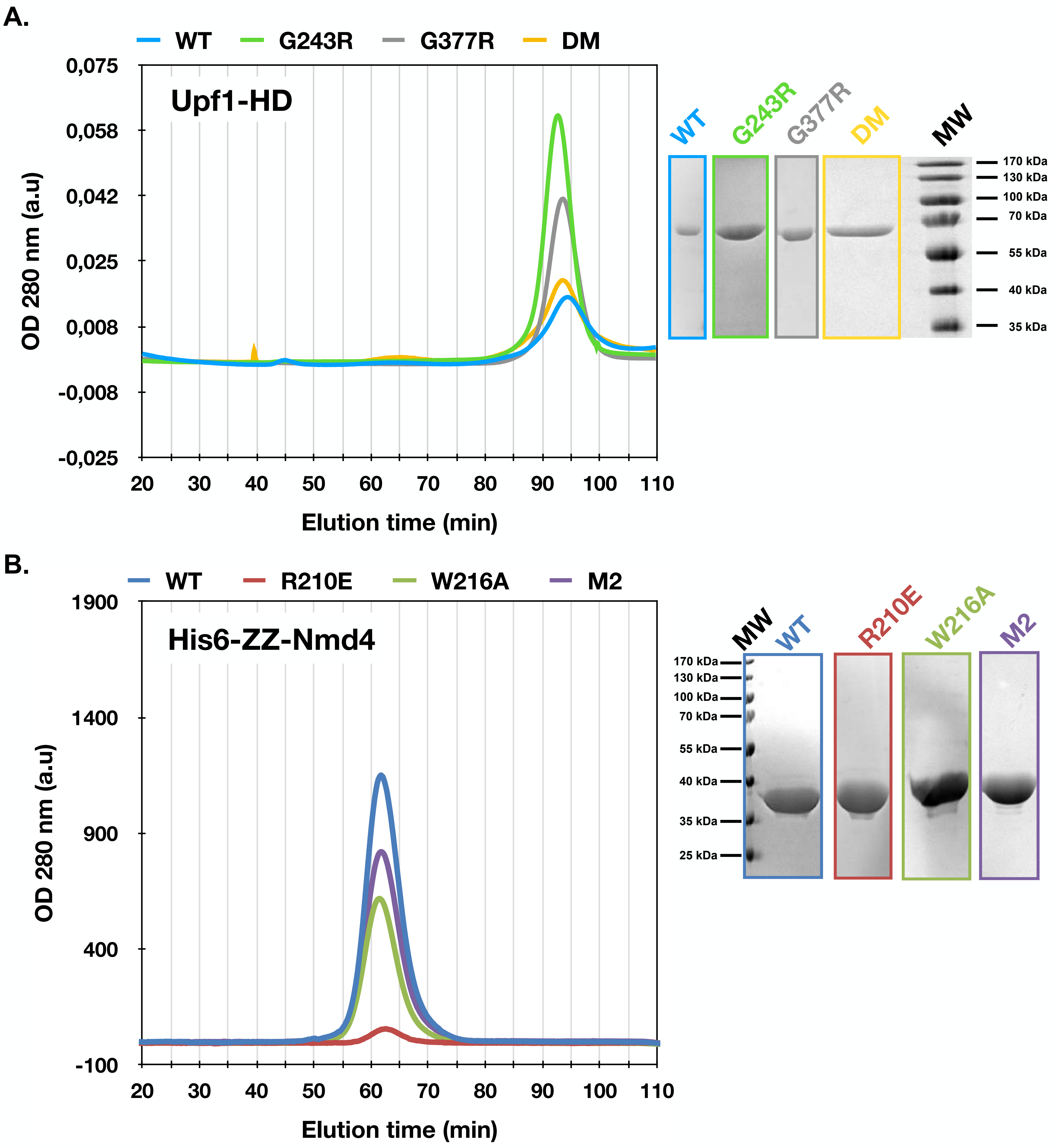

S7 Figure : Gel filtration chromatograms of *S. cerevisiae* Upf1-HD (A) or Nmd4 (B) proteins used in our *in vitro* assays.

A. Size exclusion chromatography (S200 16/60) profiles obtained for pure Upf1-HD WT and mutant proteins, showing very similar retention times for these different proteins. The Coomassie stained SDS-PAGE analysis of the proteins present in the main peak is shown for every protein (WT and mutants) to show the purity of the final proteins. MW : Molecular weight ladder.

B. Size exclusion chromatography (S75 16/60) profiles obtained for pure His_6-ZZ-Nmd4 WT and mutant proteins, showing very similar retention times for these different proteins. The Coomassie stained SDS-PAGE analysis of the proteins present in the main peak is shown for every protein (WT and mutants) to show the purity of the final proteins. MW : Molecular weight ladder.

The data underlying this figure can be found in S7 Data.
