# Supplementary material for: Structure of the Nmd4-Upf1 complex supports conservation of the nonsense-mediated mRNA decay pathway between yeast and humans
Source: PLoS Biol. 2024 Sep 27;22(9):e3002821. doi: 10.1371/journal.pbio.3002821 (PMC11463774; doi:10.1371/journal.pbio.3002821)
Supplement: S8 Fig — The amounts of expressed UPF1 variants from plasmids were estimated by immunoblot against the N-terminal TAP tag. Each sample was diluted to one half and one fourth, to estimate the quantitative aspect of the immunoblot. (PDF) [file pbio.3002821.s008.pdf]

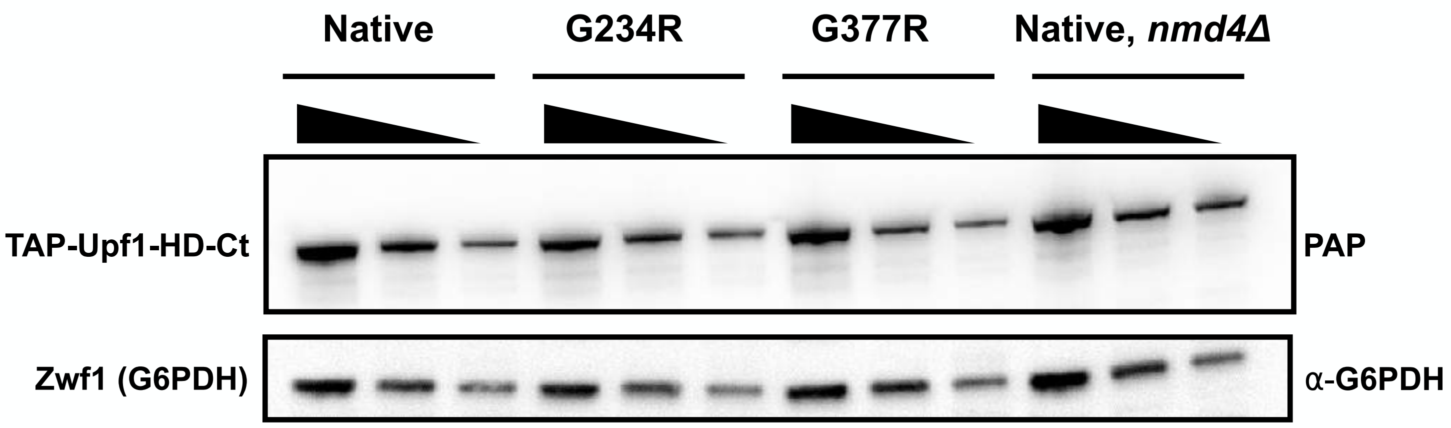

S8 Figure : Control figure (immunoblot TAP-Upf1 variants).
The amounts of expressed UPF1 variants from plasmids were estimated by immunoblot against the N-terminal TAP tag. Each sample was diluted to one half and one fourth, to estimate the quantitative aspect of the immunoblot.
