# Supplementary material for: Structure of the Nmd4-Upf1 complex supports conservation of the nonsense-mediated mRNA decay pathway between yeast and humans
Source: PLoS Biol. 2024 Sep 27;22(9):e3002821. doi: 10.1371/journal.pbio.3002821 (PMC11463774; doi:10.1371/journal.pbio.3002821)
Supplement: S9 Fig — (A) Superposition of the 5 AF3 models of the UPF1-HD/SMG6-[398–494] complex. For the sake of clarity, only 1 UPF1-HD model is shown in blue. The SMG6 fragments are colored differently depending on the models. The coordinates of the 5 AF3 models are provided as S1–S5 Files. (B) Overview of the best AF3 model of the human SMG6 [398–494] region bound to UPF1-HD domain, colored by pLDDT values. For the sake of clarity, only SMG6 residues 438 to 480 are shown. (C) Detailed view of the interface between human UPF1-HD (same color code as Fig 1B) and SMG6-[398–494] (colored by pLDDT values) in the best AF3 model. Residues from human SMG6 shown as sicks are in orange and underlined. (D) Predicted aligned error (PAE) plot of the prediction of the UPF1-HD/SMG6-[398–494] complex. This panel was generated using the PAE Viewer website (https://subtiwiki.uni-goettingen.de/v4/paeViewerDemo), the S1 File as structure file and S6 File as scores file. (E) Superposition of AF3 model of the human SMG6 [398–494] region (orange) bound to UPF1-HD domain (omitted for the sake of clarity) onto the yeast Nmd4/Upf1-HD crystal structure (same color code as Fig 1B). The side chains of R210 and W216 from Nmd4 (underlined labels) and R448 and W456 (labels underlined and in italics) of SMG6 are shown as sticks. The Cα atoms of yeast Upf1 Gly243 and Gly377 are shown as spheres. (PDF) [file pbio.3002821.s009.pdf]

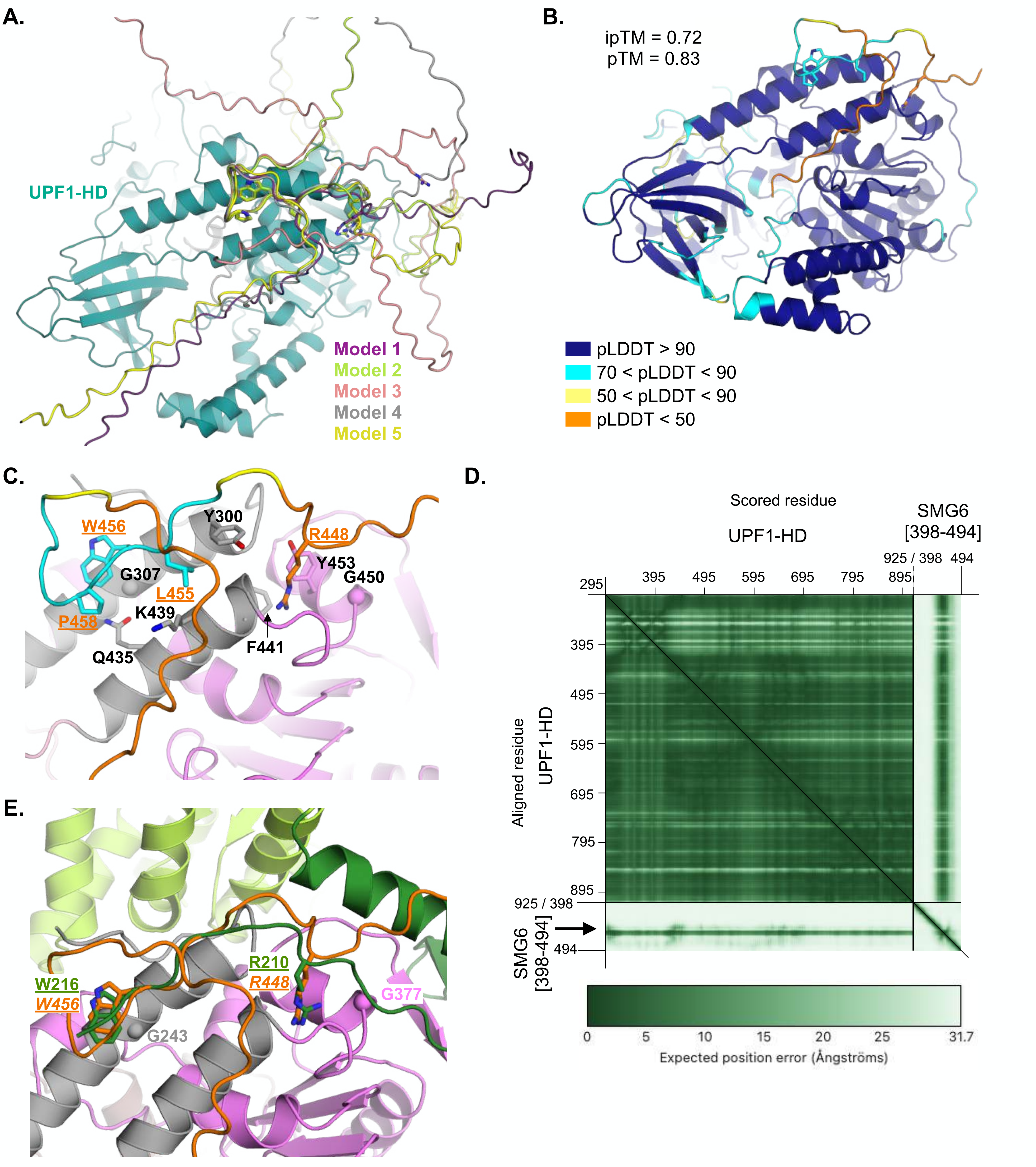

S9 Figure : AlphaFold3 model of the complex between human SMG6 and UPF1-HD.

A. Superposition of the five AF3 models of the UPF1-HD/SMG6-[398-494] complex. For the sake of clarity, only one UPF1-HD model is shown in blue. The SMG6 fragments are colored differently depending on the models.

B. Overview of the best AF3 model of the human SMG6 [398-494] region bound to UPF1-HD domain, colored by pLDDT values. For the sake of clarity, only SMG6 residues 438 to 480 are shown.

C. Detailed view of the interface between human UPF1-HD (same color code as Fig. 1B) and SMG6-[398-494] (colored by pLDDT values) in the best AF3 model. Residues from human SMG6 shown as sicks are in orange and underlined.

D. Predicted aligned error (PAE) plot of the prediction of the UPF1-HD/SMG6-[398-494] complex.

E. Superposition of AF3 model of the human SMG6 [398-494] region (orange) bound to UPF1-HD domain (omitted for the sake of clarity) onto the yeast Nmd4/Upf1-HD crystal structure (same color code as Fig. 1B). The side chains of R210 and W216 from Nmd4 (underlined labels) and R448 and W456 (labels underlined and in italics) of SMG6 are shown as sticks. The Cα atoms of yeast Upf1 Gly243 and Gly377 are shown as spheres.
