# Supplementary material for: Structure of the Nmd4-Upf1 complex supports conservation of the nonsense-mediated mRNA decay pathway between yeast and humans
Source: PLoS Biol. 2024 Sep 27;22(9):e3002821. doi: 10.1371/journal.pbio.3002821 (PMC11463774; doi:10.1371/journal.pbio.3002821)
Supplement: S10 Fig — Total extracts were loaded on SDS-PAGE and proteins were detected by western blot using the indicated antibodies. (PDF) [file pbio.3002821.s010.pdf]

Empty plasmid	+	+				
shRNA against endogenous SMG6		+	+	+	+	+
RNAi resistant HA-SMG6 WT			+			
RNAi resistant HA-SMG6 M2				+		
RNAi resistant HA-SMG6 M5					+	
RNAi resistant HA-SMG6 M7						+

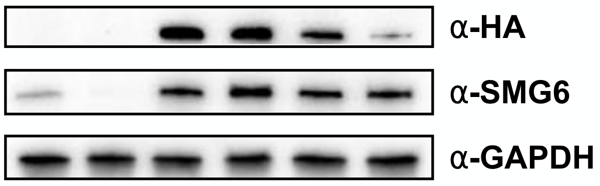

S10 Figure : Control figure showing the silencing of endogenous SMG6 and the rescue by the expression of shRNA-resistant variants of SMG6. Total extracts were loaded on SDS-PAGE and proteins were detected by western blot using the indicated antibodies.
