# Supplementary material for: Structure of the Nmd4-Upf1 complex supports conservation of the nonsense-mediated mRNA decay pathway between yeast and humans
Source: PLoS Biol. 2024 Sep 27;22(9):e3002821. doi: 10.1371/journal.pbio.3002821 (PMC11463774; doi:10.1371/journal.pbio.3002821)
Supplement: S1 Raw Images — (PDF) [file pbio.3002821.s025.pdf]

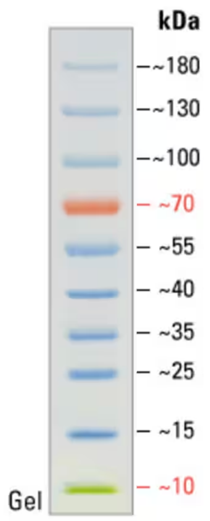

Prestained PageRulerTM
(ThermoFisher; #26616)

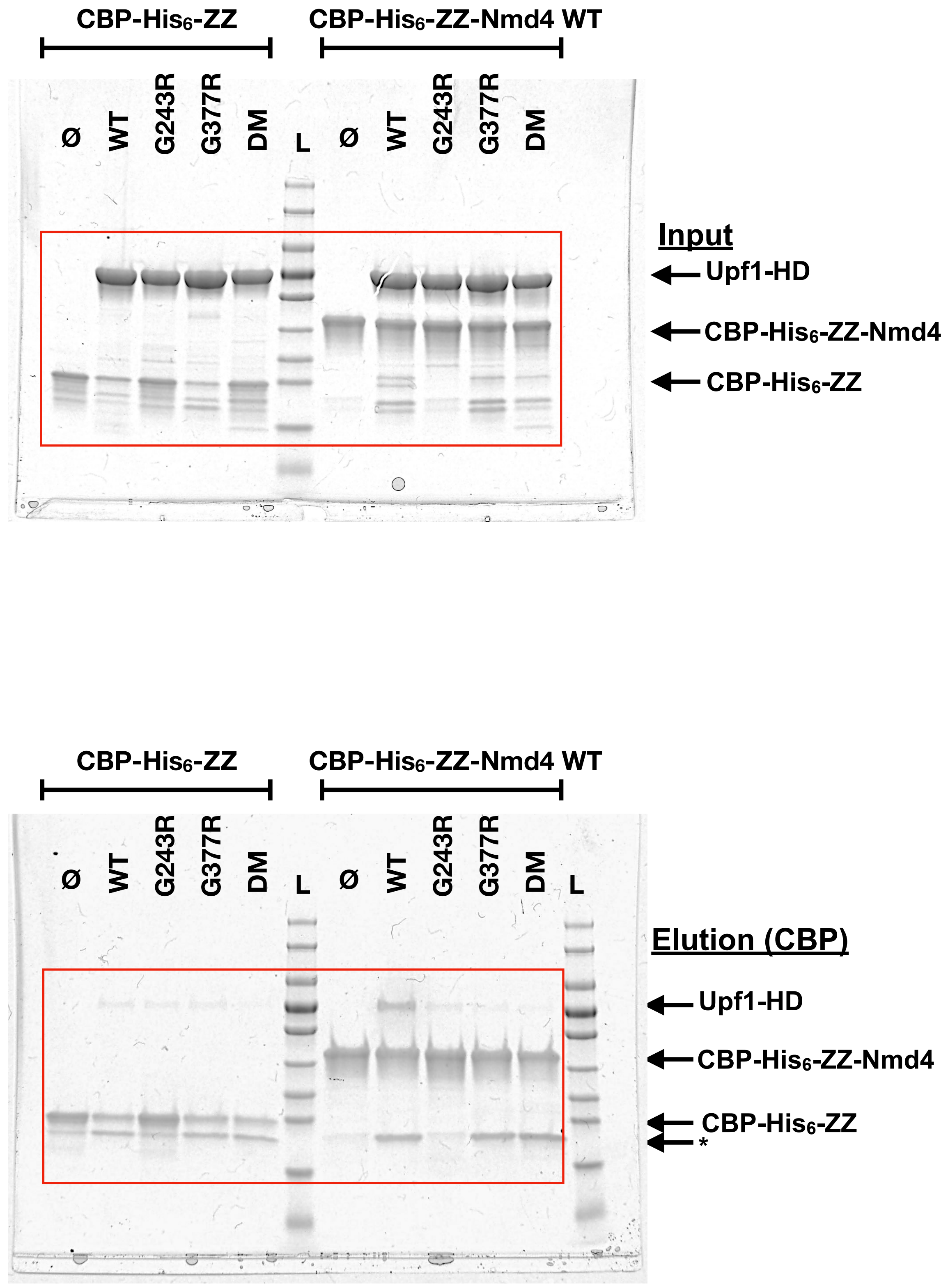

Figure 3C

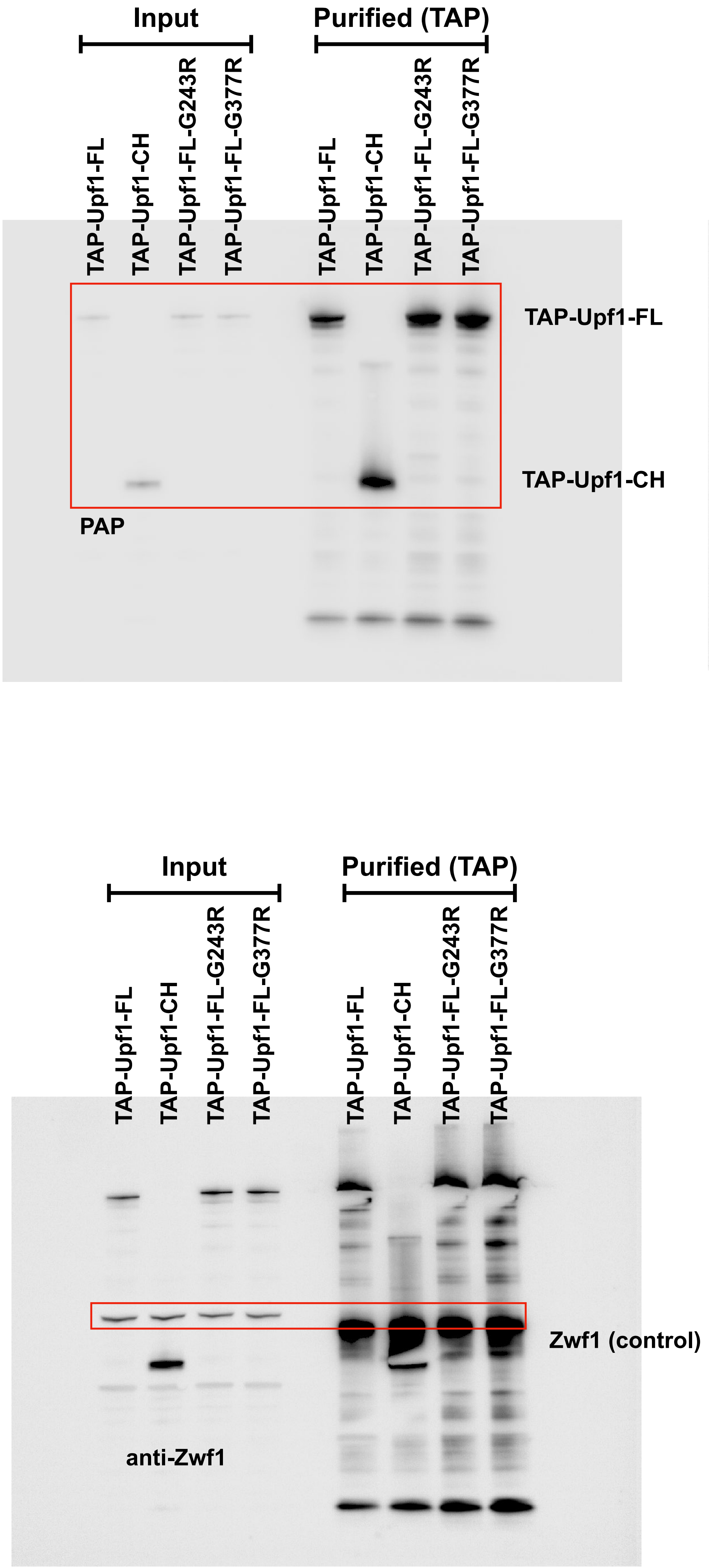
Input
Purified (TAP)
TAP-Upf1-FL
TAP-Upf1-CH
TAP-Upf1-FL-G243R
TAP-Upf1-FL-G377R
TAP-Upf1-FL
TAP-Upf1-CH
TAP-Upf1-FL-G243R
TAP-Upf1-FL-G377R
TAP-Upf1-FL
TAP-Upf1-CH
PAP
Input
Purified (TAP)
TAP-Upf1-FL
TAP-Upf1-CH
TAP-Upf1-FL-G243R
TAP-Upf1-FL-G377R
TAP-Upf1-FL
TAP-Upf1-CH
TAP-Upf1-FL-G243R
TAP-Upf1-FL-G377R
Nmd4-FL-HA
anti-HA
Input
Purified (TAP)
TAP-Upf1-FL
TAP-Upf1-CH
TAP-Upf1-FL-G243R
TAP-Upf1-FL-G377R
TAP-Upf1-FL
TAP-Upf1-CH
TAP-Upf1-FL-G243R
TAP-Upf1-FL-G377R
Zwf1 (control)
anti-Zwf1

Figure 3D

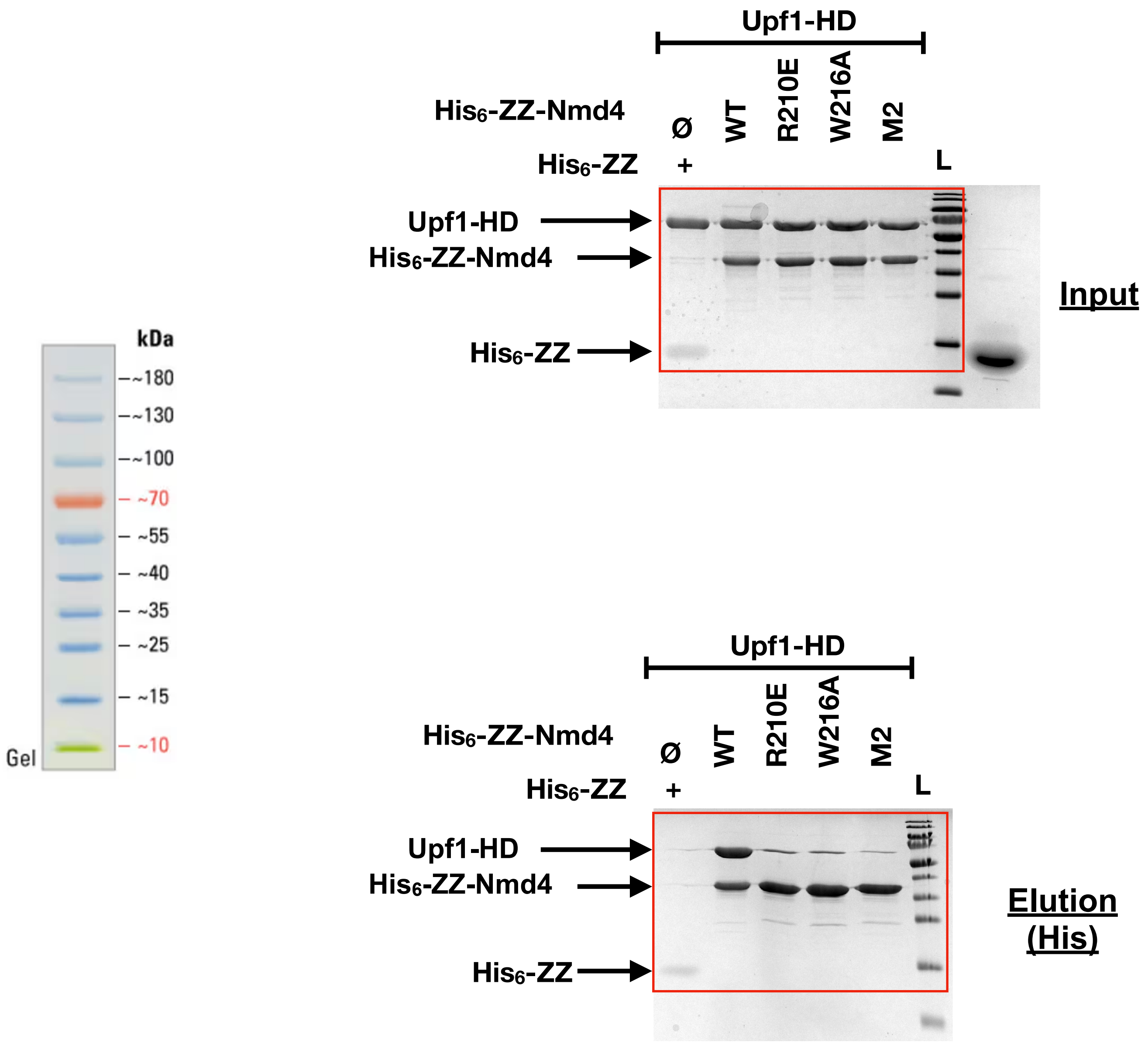
kDa
~180
~130
~100
~70
~55
~40
~35
~25
~15
~10
Gel
Upf1-HD
His6-ZZ-Nmd4
Ø
WT
R210E
W216A
M2
His6-ZZ
+
L
Upf1-HD
His6-ZZ-Nmd4
His6-ZZ
Input
Upf1-HD
His6-ZZ-Nmd4
Ø
WT
R210E
W216A
M2
His6-ZZ
+
L
Upf1-HD
His6-ZZ-Nmd4
His6-ZZ
Elution
(His)

Figure 3F

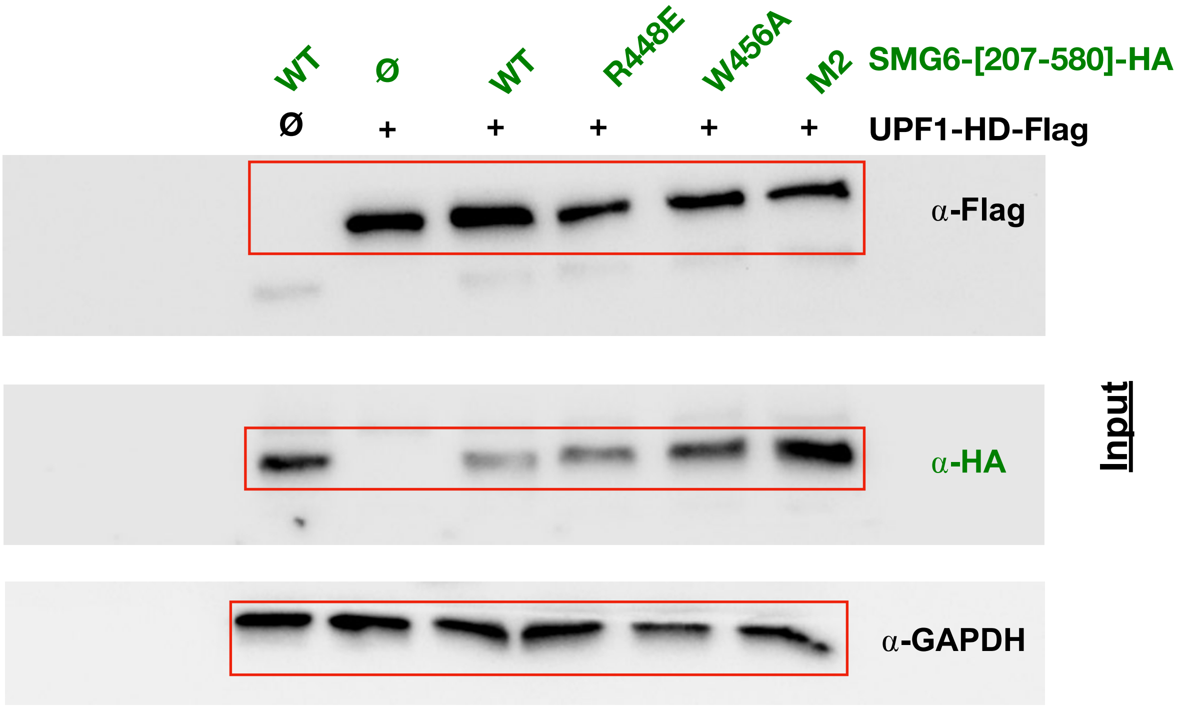
WT
Ø
WT
R448E
W456A
M2
SMG6-[207-580]-HA
Ø + + + + +
UPF1-HD-Flag
α-Flag
α-HA
α-GAPDH
Input

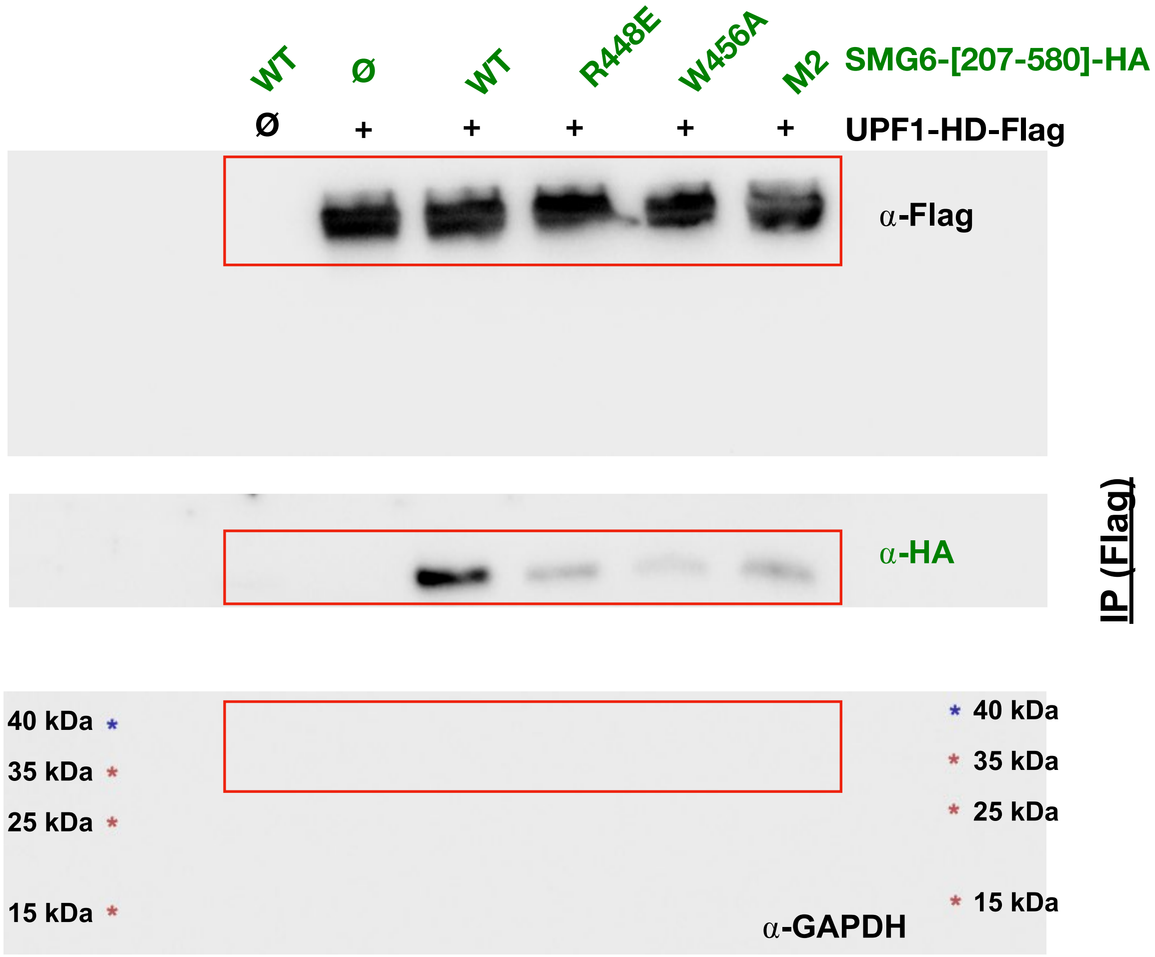
WT
Ø
WT
R448E
W456A
M2
SMG6-[207-580]-HA
Ø + + + + +
UPF1-HD-Flag
α-Flag
α-HA
IP (Flag)
40 kDa
35 kDa
25 kDa
15 kDa
40 kDa
35 kDa
25 kDa
15 kDa
α-GAPDH

Figure 4B

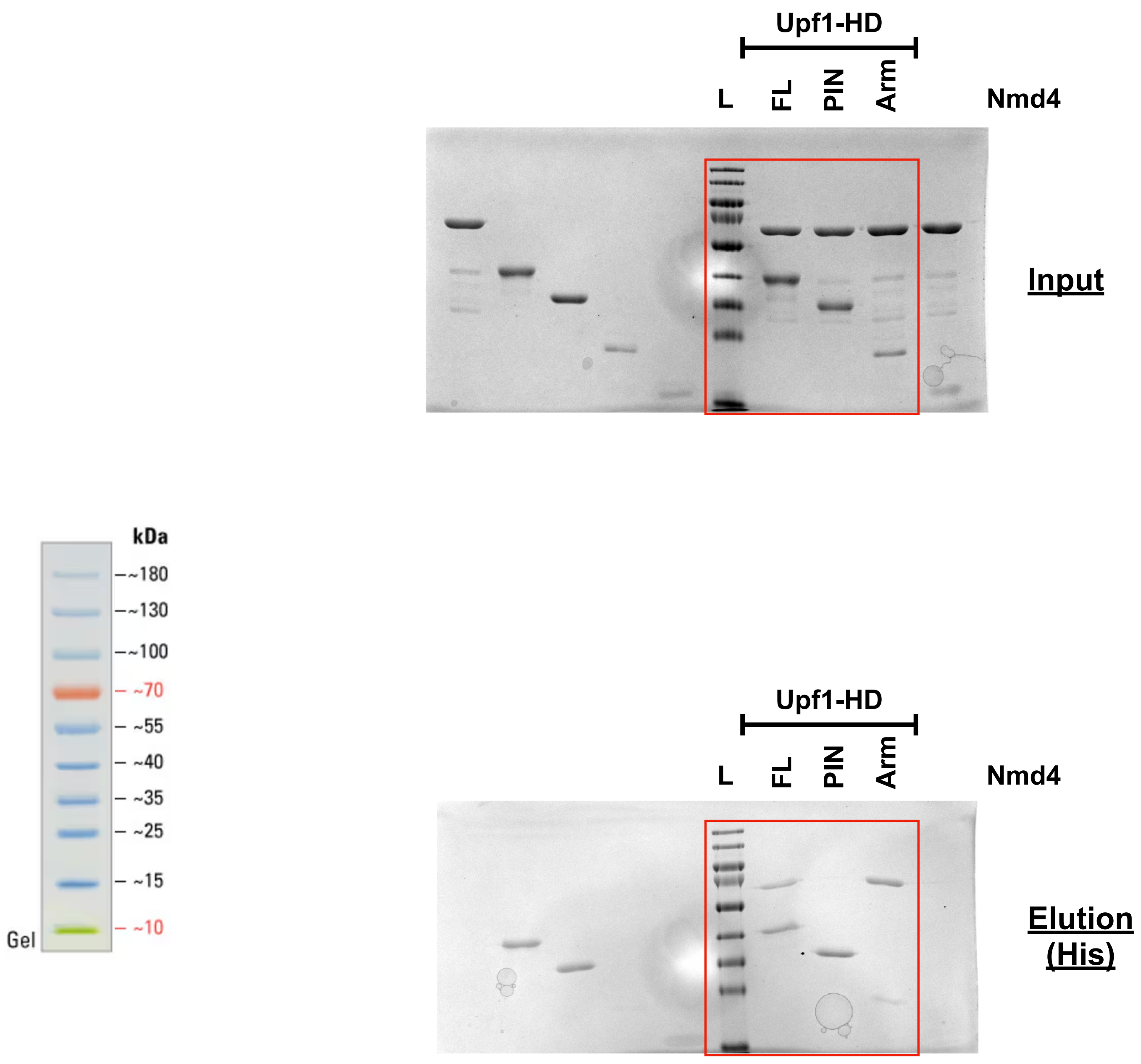
Upf1-HD
L
FL
PIN
Arm
Nmd4
Input
kDa
~180
~130
~100
~70
~55
~40
~35
~25
~15
~10
Gel
Upf1-HD
L
FL
PIN
Arm
Nmd4
Elution
(His)

S5A Figure

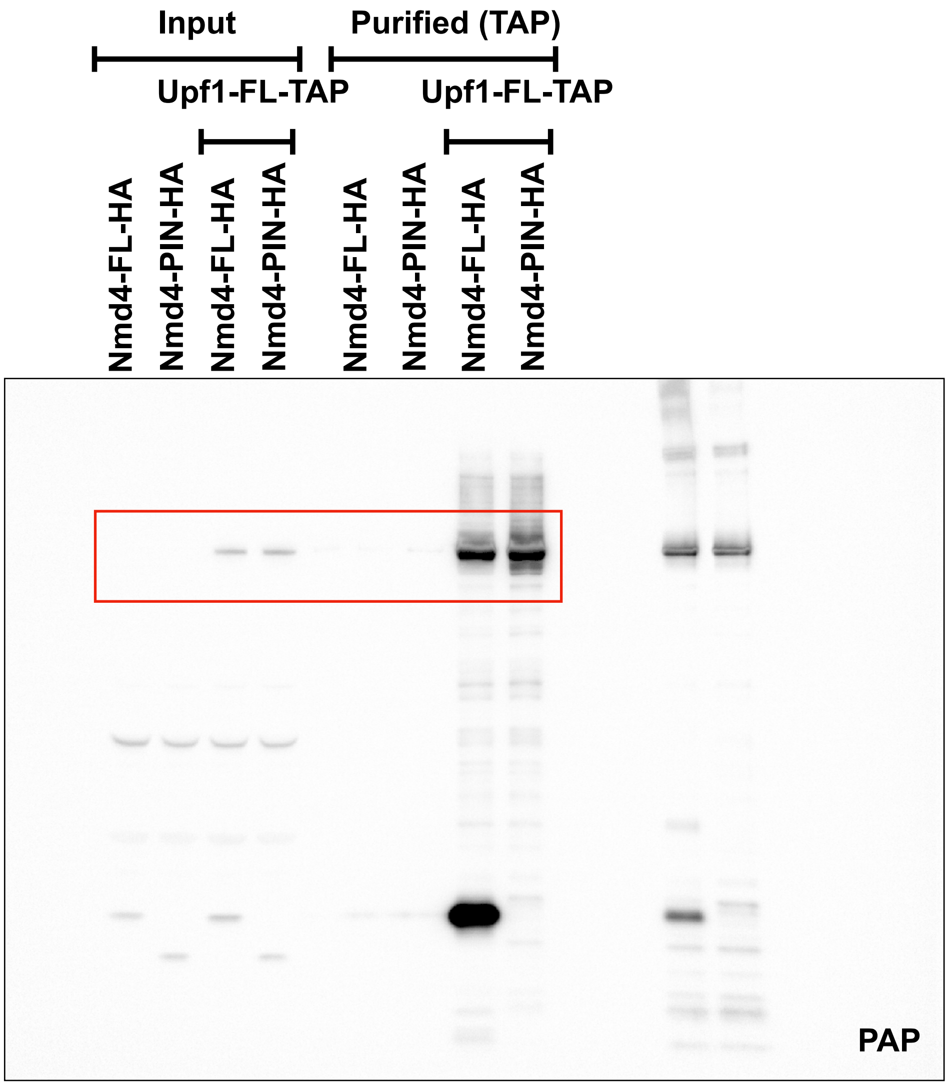
Input
Purified (TAP)
Upf1-FL-TAP
Upf1-FL-TAP
Nmd4-FL-HA
Nmd4-PIN-HA
Nmd4-FL-HA
Nmd4-PIN-HA
Nmd4-FL-HA
Nmd4-PIN-HA
Nmd4-FL-HA
Nmd4-PIN-HA
PAP

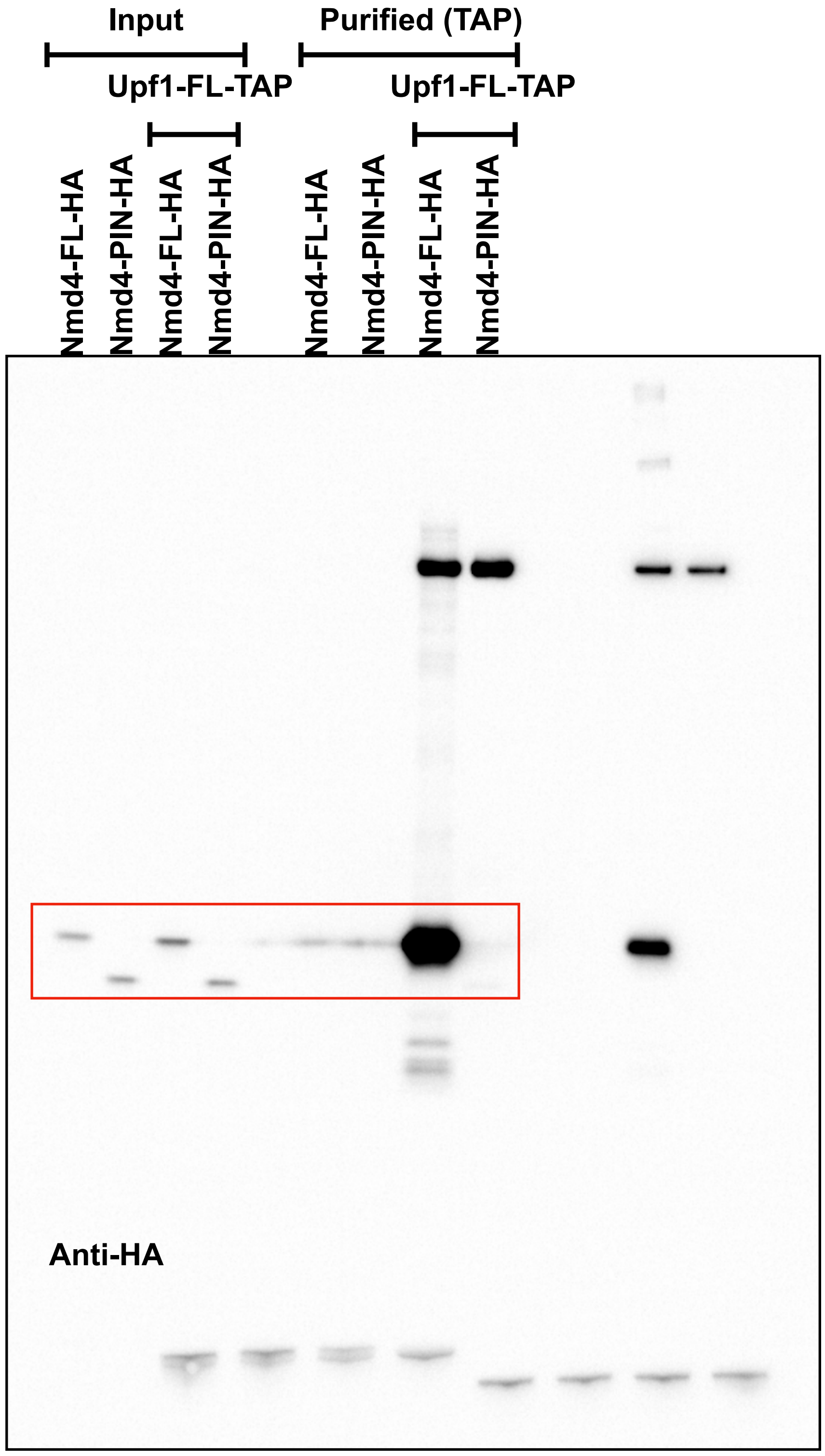
Input
Purified (TAP)
Upf1-FL-TAP
Upf1-FL-TAP
Nmd4-FL-HA
Nmd4-PIN-HA
Nmd4-FL-HA
Nmd4-PIN-HA
Nmd4-FL-HA
Nmd4-PIN-HA
Nmd4-FL-HA
Nmd4-PIN-HA
Anti-HA

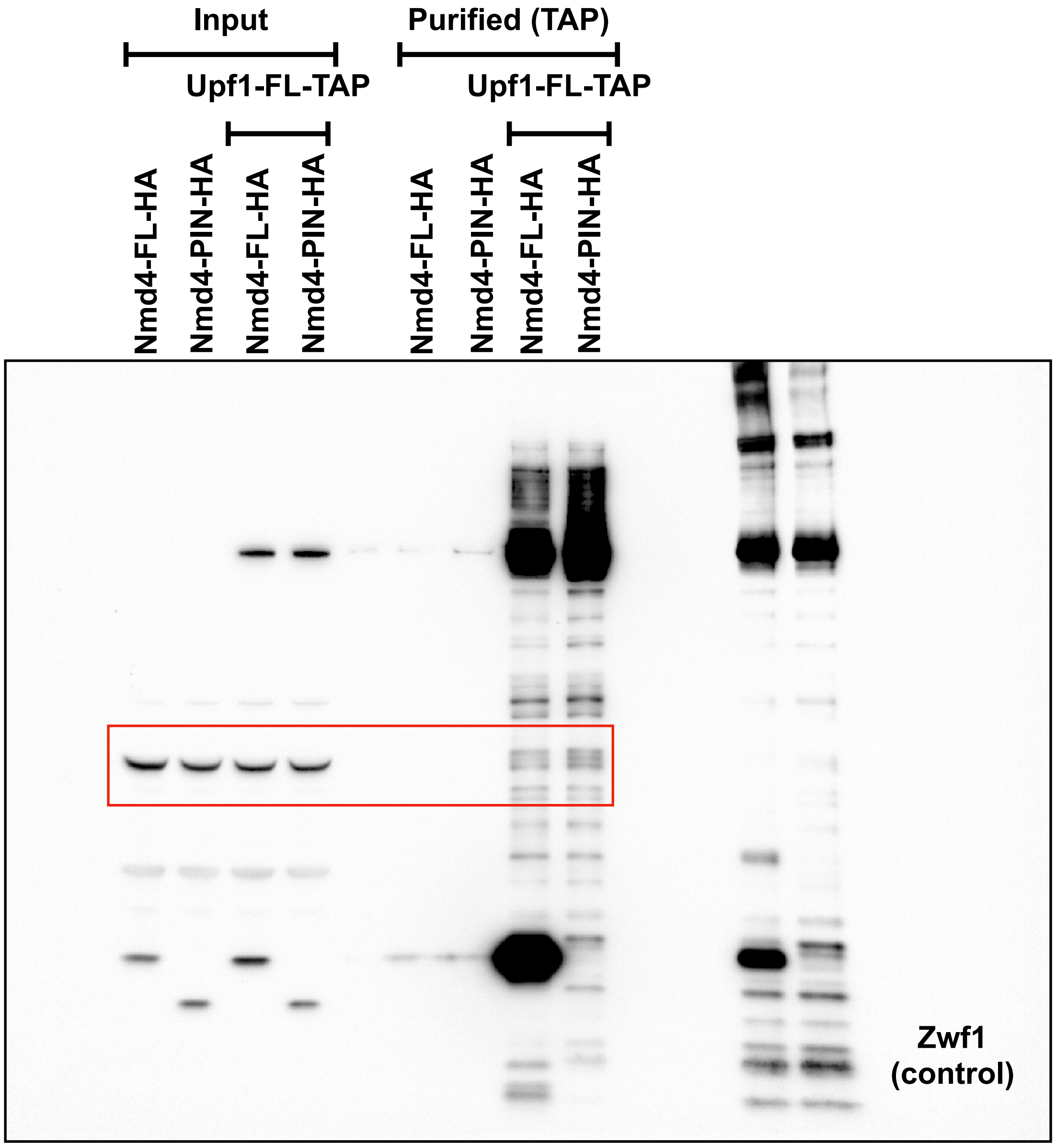
Input
Purified (TAP)
Upf1-FL-TAP
Upf1-FL-TAP
Nmd4-FL-HA
Nmd4-PIN-HA
Nmd4-FL-HA
Nmd4-PIN-HA
Nmd4-FL-HA
Nmd4-PIN-HA
Nmd4-FL-HA
Nmd4-PIN-HA
Zwf1
(control)

S5B Figure

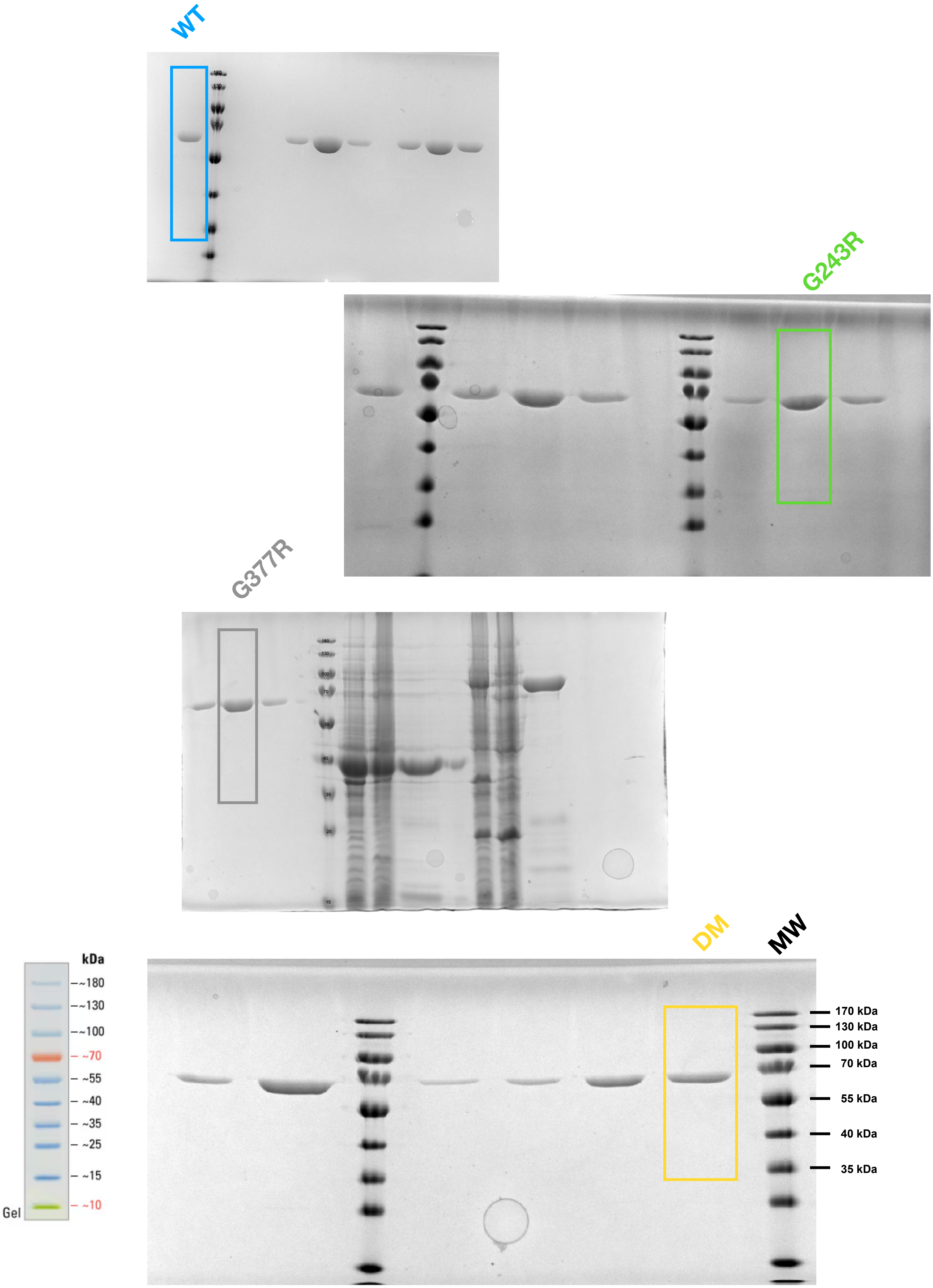
WT
G243R
G377R
DM
MW
kDa
~180
~130
~100
~70
~55
~40
~35
~25
~15
~10
Gel
170 kDa
130 kDa
100 kDa
70 kDa
55 kDa
40 kDa
35 kDa

S7A Figure

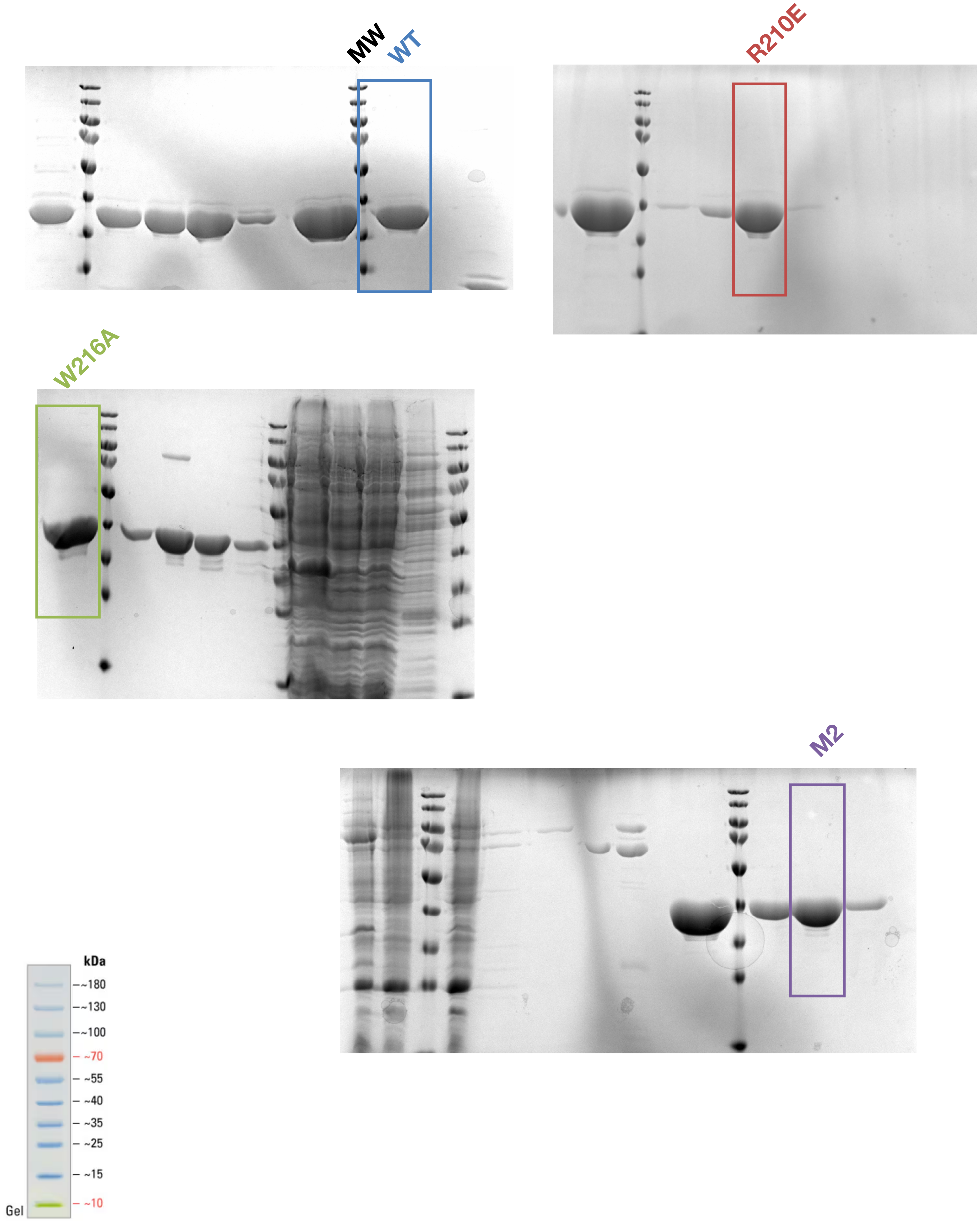

S7B Figure

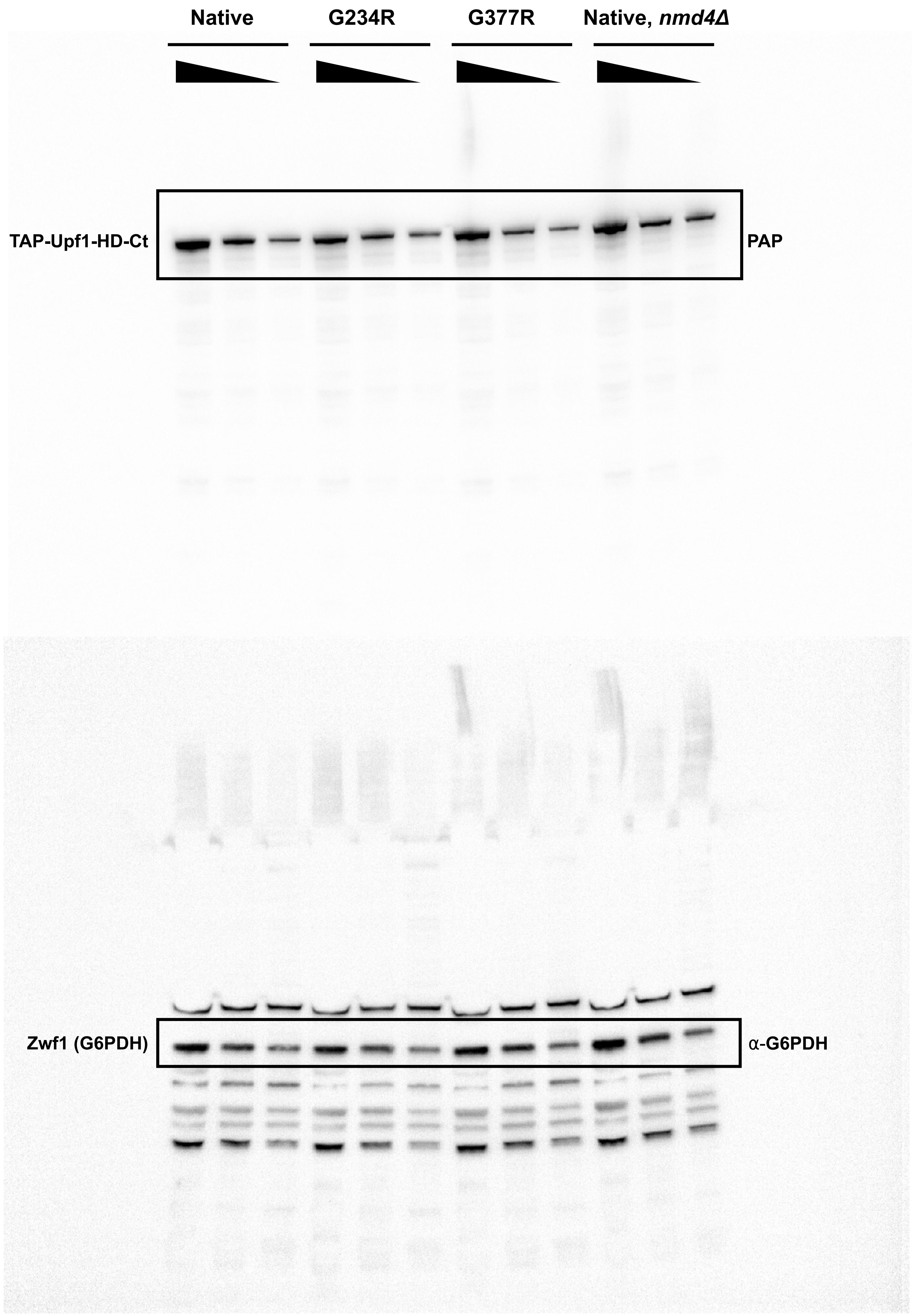
Native
G234R
G377R
Native, nmd4Δ
TAP-Upf1-HD-Ct
PAP
Zwf1 (G6PDH)
α-G6PDH

S8 Figure

Empty plasmid	+	+				
shRNA against endogenous SMG6		+	+	+	+	+
RNAi resistant HA-SMG6 WT			+			
RNAi resistant HA-SMG6 M2				+		
RNAi resistant HA-SMG6 M5					+	
RNAi resistant HA-SMG6 M7						+

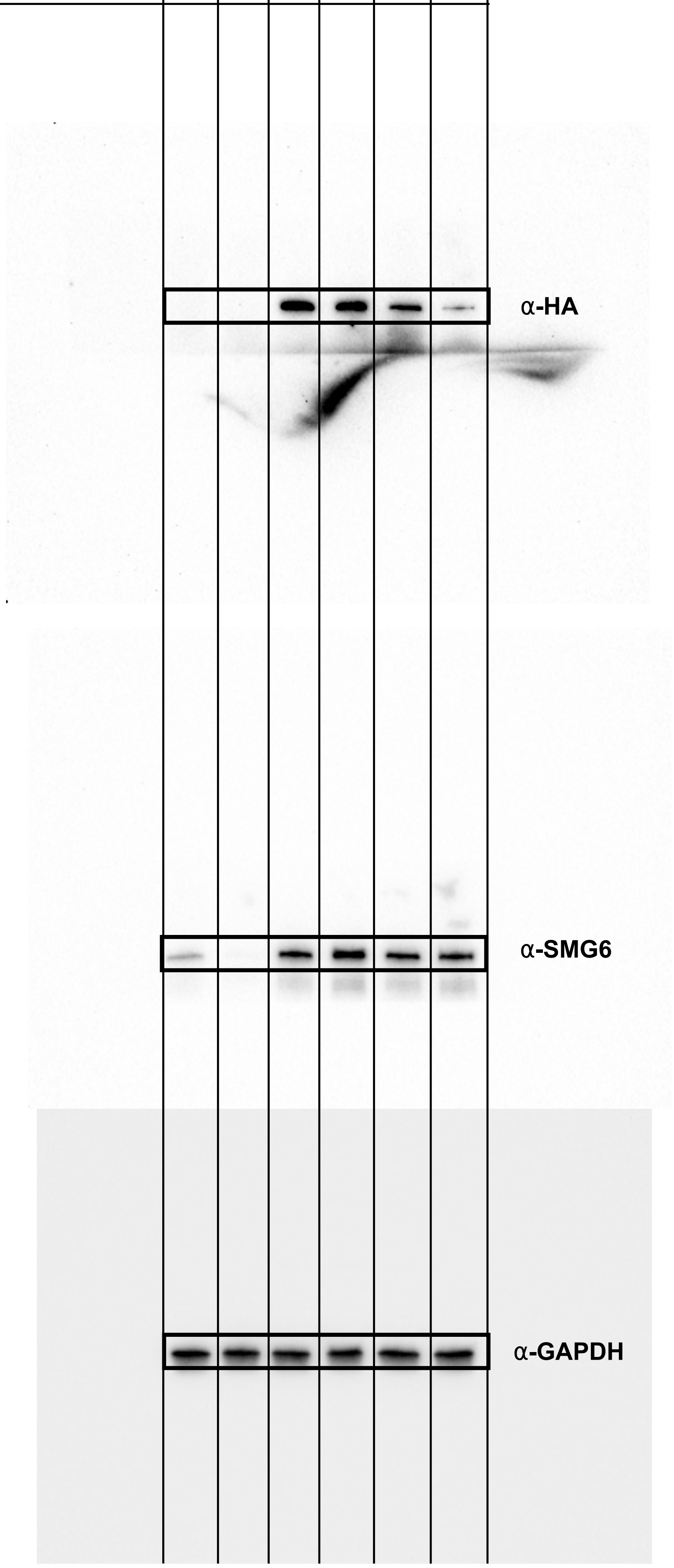

S10 Figure
