# Supplementary material for: Structure of the Nmd4-Upf1 complex supports conservation of the nonsense-mediated mRNA decay pathway between yeast and humans
Source: PLoS Biol. 2024 Sep 27;22(9):e3002821. doi: 10.1371/journal.pbio.3002821 (PMC11463774; doi:10.1371/journal.pbio.3002821)
Supplement: S4 Fig — Strictly conserved residues are in white on a black background. Partially conserved amino acids are shown in bold. Secondary-structure elements, as observed in the crystal structure of the S. cerevisiae Upf1-HD protein bound to Nmd4, are shown above the alignment. Positions involved in the interface with Upf1-HD are indicated by black filled spheres below the alignment. The yeast Upf1 residues G243 and G377 mutated in this study are boxed in red. Domain boundaries are indicated above the alignment using the color code defined in Fig 1A. This figure was generated using the ENDscript server [76]. (PDF) [file pbio.3002821.s004.pdf]

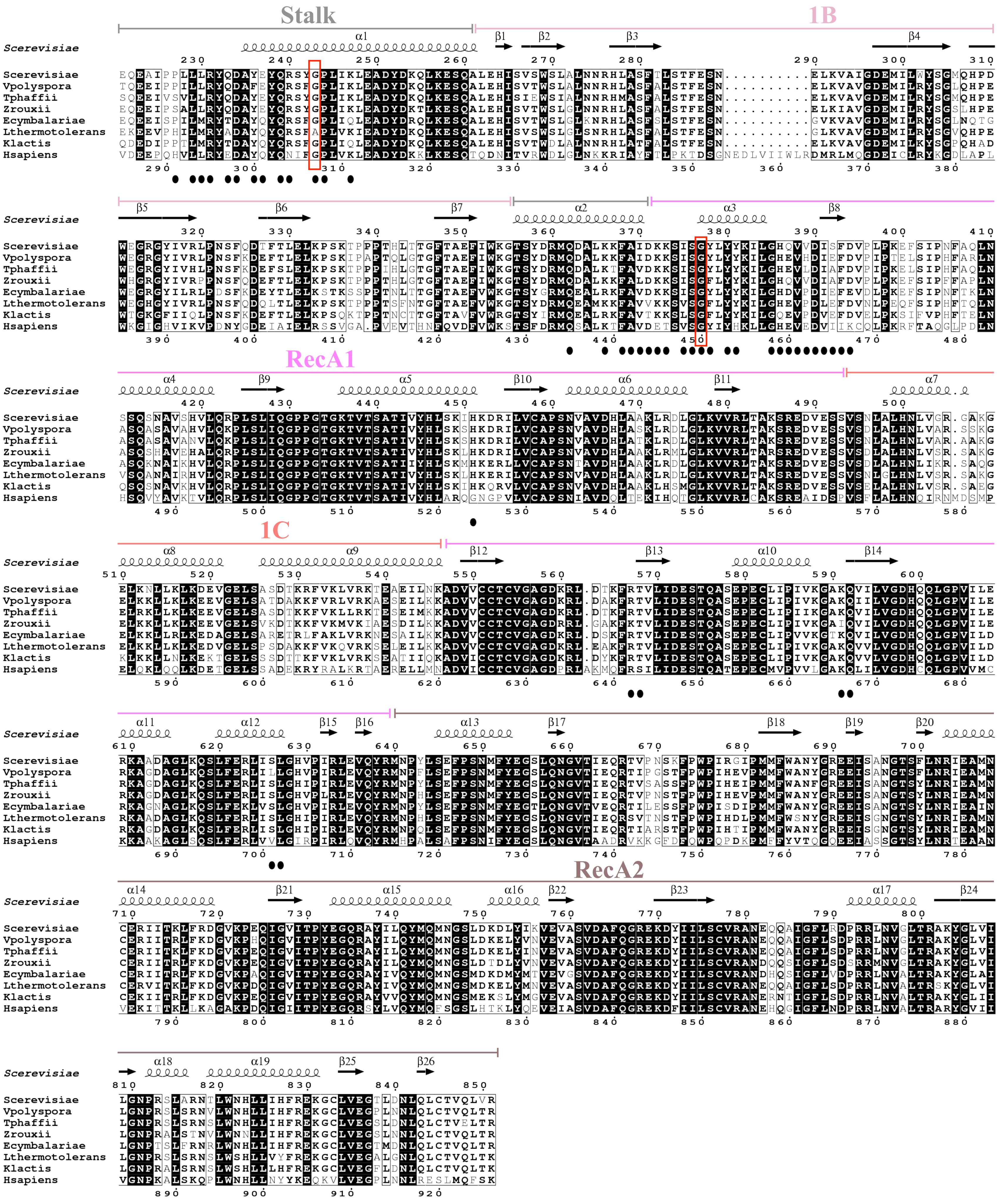

S4 Figure : Multiple sequence alignment of Upf1 proteins.

Strictly conserved residues are in white on a black background. Partially conserved amino acids are shown in bold. Secondary-structure elements, as observed in the crystal structure of the *S. cerevisiae* Upf1-HD protein bound to Nmd4, are shown above the alignment. Positions involved in the interface with Upf1-HD are indicated by black filled spheres below the alignment. The yeast Upf1 residues G243 and G377 mutated in this study are boxed in red. Domain boundaries are indicated above the alignment using the color code defined in Fig. 1A. This figure was generated using the ENDscript server (https://espript.ibcp.fr/ESPript/cgi-bin/ESPript; [76]).
