# Supplementary material for: Structure of the Nmd4-Upf1 complex supports conservation of the nonsense-mediated mRNA decay pathway between yeast and humans
Source: PLoS Biol. 2024 Sep 27;22(9):e3002821. doi: 10.1371/journal.pbio.3002821 (PMC11463774; doi:10.1371/journal.pbio.3002821)
Supplement: Attachment — Submitted filename: Response-reviewers-PBIOLOGY-D-24-01058R1_202400802.pdf [file pbio.3002821.s034.pdf]

We would like to thank the reviewers and the academic editor for their comments on our manuscript. In this revised version, we have taken into consideration their very positive and insightful comments.

REVIEWS:

Reviewer #1: The authors have adequately addressed the points I raised in my previous review. I believe that the manuscript is suitable for publication.

My remaining concern is about the included Alphafold model in Fig EV6.
This figure also needs to show the Alphafold confidence scores, otherwise readers are unable to appreciate whether the model can be trusted.
- One panel should show the model colored according to the pLDDT score.
- If the SMG6 region binding to UPF1 is not clearly visible in the pLDDT-coloured figure, it should also be shown on its own with highlighting UPF1-binding residues as sticks (pLDDT- colored).
- A PAE diagram for the complex prediction should also be shown
- A panel showing the predicted interaction details could be shown.

We would like to thank reviewer #1 for this comment, which allowed us to improve our description of the AlphaFold model for the complex formed by human UPF1 helicase domain and the SMG6 region encompassing residues 398-494 that we identify as UPF1 binding motif in this study. Since a new version of AlphaFold (v3; AF3; PMID : 38718835) has been released very recently and is supposed to provide more accurate models of protein-protein complexes, we have generated five models for the complex between human UPF1-HD and SMG6-[398-494]. All five models are largely consistent between each others and we have decided to focus on the model with the highest ipTM (0.72; interface predicted template modeling) score. Figure EV6 has been updated accordingly and now presents the superposition of all five models generated by AF3 (panel A), the representation of the model with the highest ipTM colored according to pLDDT values (panel B), a zoom in the proposed human UPF1-HD/SMG6 interface with some important residues (such as R448 and W456) highlighted in sticks (panel C), the PAE diagram for the complex prediction (panel D) and a comparison between the crystal structure of the yeast Upf1-HD/Nmd4 complex and the UPF1-HD/SMG6 model highlighting residues mutated in this study (panel E).
The legend for Fig. EV6 has been modified accordingly :
« **Figure EV6 : AlphaFold3 model of the complex between human SMG6 and UPF1-HD.**
A. Superposition of the five AF3 models of the UPF1-HD/SMG6-[398-494] complex. For the sake of clarity, only one UPF1-HD model is shown in blue. The SMG6 fragments are colored differently depending on the models.
B. Overview of the best AF3 model of the human SMG6 [398-494] region bound to UPF1-HD domain, colored by pLDDT values. For the sake of clarity, only SMG6 residues 438 to 480 are shown.
C. Detailed view of the interface between human UPF1-HD (same color code as Fig. 1B) and SMG6-[398-494] (colored by pLDDT values) in the best AF3 model. Residues from human SMG6 shown as sicks are in orange and underlined.
D. Predicted aligned error (PAE) plot of the prediction of the UPF1-HD/SMG6-[398-494] complex.
E. Superposition of AF3 model of the human SMG6 [398-494] region (orange) bound to UPF1-HD domain (omitted for the sake of clarity) onto the yeast Nmd4/Upf1-HD crystal structure (same color code as Fig. 1B). The side chains of R210 and W216 from Nmd4 (underlined labels) and R448 and

W456 (labels underlined and in italics) of SMG6 are shown as sticks. The Cα atoms of yeast Upf1 Gly243 and Gly377 are shown as spheres. »

The dedicated section in the material and methods has also been updated accordingly and now reads : « *Generation of the human UPF1-HD/SMG6 model*

Five models of the 3D structure of human UPF1-HD (region 295-925) bound to SMG6 region 398-494 (*i.e.* containing the conserved region 445-465) were generated using the recently launched version of AlphaFold program (version 3; AF3), with the seed auto option as defined in the AlphaFold server (https://alphafoldserver.com/; [55]). »

Reviewer #2: The authors have addressed most of my comments.

With regard to the limitation highlighted earlier, please provide a brief discussion of the consistency of phosphorylation-independent binding versus the inconsistency of nuclease activity.

To address this comment, we have added a short paragraph, which also mention the recently described identification of a short linear motif located upstream of the SMG6 « arm-like » motif, and which interacts with UPF1 CH domain (PMID : 38709891). This brings independent support to the hypothesis that phosphorylation-independent interactions between UPF1 and SMG6 are important.

The following text has been added : « SMG6 clearly emerges as a protein with multiple binding sites for UPF1 since while this article was under revision, a short linear motif (amino acids 406-413) located just upstream of the « arm-like » motif (amino acids 445-465) identified in our study, was shown to interact with the CH domain from human UPF1 (Fig. 4D; [63]). This motif competes with UPF2 to interact with UPF1 CH, further supporting the evolutionary conserved existence of two NMD complexes assembled around UPF1, namely the UPF1-23 and the UPF1-effector complex as previously shown in yeast [45]. Altogether, this supports that the conserved phosphorylation-independent binding mode between Upf1 and Nmd4 in yeasts and UPF1/SMG6 in metazoa might be more important for NMD than the nuclease activity since the catalytic residues are absent in the PIN domain from fungal Nmd4 proteins. »

Please revise the sentence "but also that a physical interaction between Upf1-HD and the PIN domain exists in vitro" to "might exist ».

This has been done.

COMMENTS FROM THE ACADEMIC EDITOR

1. This work heavily relies on the use of in vitro assays to determine the role of Nmd4-Upf1 protein-protein interactions on NMD function (ATPase assays in Figure 2/3, binding assays in Figure 3/4). Though it is encouraging, for example, that Nmd4 FL, PIN, and Arm purified proteins differentially promote Upf1 ATPase activity in Figure 1B, it is concerning that all additions increase assay output. This is especially the case given that the Nmd4 PIN domain was not shown to bind Upf1 yet still increases its ATPase activity. A control for total protein mass in the ATPase assay should be included to address this possible confounding effect, perhaps with a relevant protein without known Upf1-HD binding.

We agree with the reviewer that it was surprising to detect a stimulation of Upf1-HD ATPase activity by Nmd4 PIN domain. As additional control, we have incubated Upf1-HD with the His-ZZ tag present in the various Nmd4 constructs used in this experiment. We have demonstrated using ITC and pull-down that this tag does not interact with Upf1-HD and hence from our point-of-view, it can be considered as the best internal control. As can be seen in the new panel B of Figure 2, the addi-

tion of His-ZZ tag to Upf1-HD does not significantly stimulates Upf1-HD ATPase activity, indicating that the enhancement observed with Nmd4 PIN domain is specific.
The text has been modified to include this control : « Similarly, the His-ZZ tag used to express the various Nmd4 construct did not activate significantly Upf1-HD activity. This indicates that the observed increase in enzymatic activity is specific to the binding of Nmd4 to Upf1-HD (Fig. 2B). »

2. If the effects seen in Figure 3E, with respect to processing of DAL7 and pre-L28, are truly dependent on Nmd4 binding to the Upf1-HD-Ct protein, deletion of Nmd4 in the background of Upf1-HD-Ct G243R and G377R should demonstrate no change from expression of these constructs in an NMD4 strain. These two controls (Upf1-HD-Ct G243R and G377R in a upf1nmd4 background) should be added to confirm that the effects are dependent on Upf1-Nmd4 binding. Furthermore, results in 3F suggest that Nmd4 R210E, W216A, and M2 should act similarly to nmd4 in the experiments from 3E, which would be important controls to validate in vivo the in vitro findings from other panels in Figure 3.

To answer this question, we measured DAL7 and pre-L28 RNA levels in a double mutant strain lacking UPF1 and NMD4, complemented either by Upf1-HD-Ct or G243R and G377R variants expressed from a plasmid. We observed similar changes in the endogenous reporter transcript levels in these three conditions when compared with the strain that expresses endogenous Nmd4 (new panel Fig. 3E). These results further support that Upf1 molecules that no longer bind Nmd4 have a lower ability to function in yeast NMD. The corresponding section of the manuscript has been modified as follow: « Importantly, the complementation by Upf1-HD-Ct is further reduced in the *upf1Δ/nmd4Δ* double mutant. In the presence of the Upf1-HD-Ct construct, the levels of DAL7 and pre-L28 NMD substrates were similar in the *upf1Δ* strains lacking Nmd4 or upon expression of Upf1-HD-Ct G243R or G377R point mutants in the *upf1Δ* or *upf1Δ/nmd4Δ* strains. Altogether, this indicates that the interaction between Nmd4 and Upf1 is important for NMD in the absence of the Upf1 CH domain, *i.e.* when the efficient recruitment of Upf2 and/or decapping factors is impaired for instance [27,53]. »

We agree with the second suggestion from the reviewer that Nmd4 mutations altering its affinity for Upf1 would be expected to affect Upf1 function. However, deletion of NMD4 alone has a very mild NMD phenotype and the range of effects seen on the levels of an excellent NMD substrate such as DAL7 is less than a factor of 2. To test the behavior of Nmd4 mutants, we cloned a region encompassing the Nmd4 coding sequence and flanking regions including the promoter and terminator into a yeast CEN vector (LEU2) and transformed the plasmids with WT, R210E, W216A and double mutant (M2) in a strain with the double deletion of UPF1 and NMD4, complemented with Upf1-HD-Cter on another CEN plasmid (URA3). Five independent cultures were used to assess the relative changes in the levels of DAL7 NMD substrate versus PGK1. As shown in the figure shown below, the M2 mutant was clearly less able to reduce DAL7 levels than the wt in four out of five experiments. In those experiments, the M2 mutant behaved similar to an empty, control, plasmid. For the single amino acid change variants, the results were intermediate. Although the complexity of the experimental system and its inherent variability are high (most probably due to the need to transfect with two plasmids in our experimental setup), the results confirmed our findings and the hypothesis put forward by the reviewer. At the moment, it is our feeling that this figure should not be included in the revised version. If you consider that it should be included as supplementary materials in this article, we would be happy to do so.

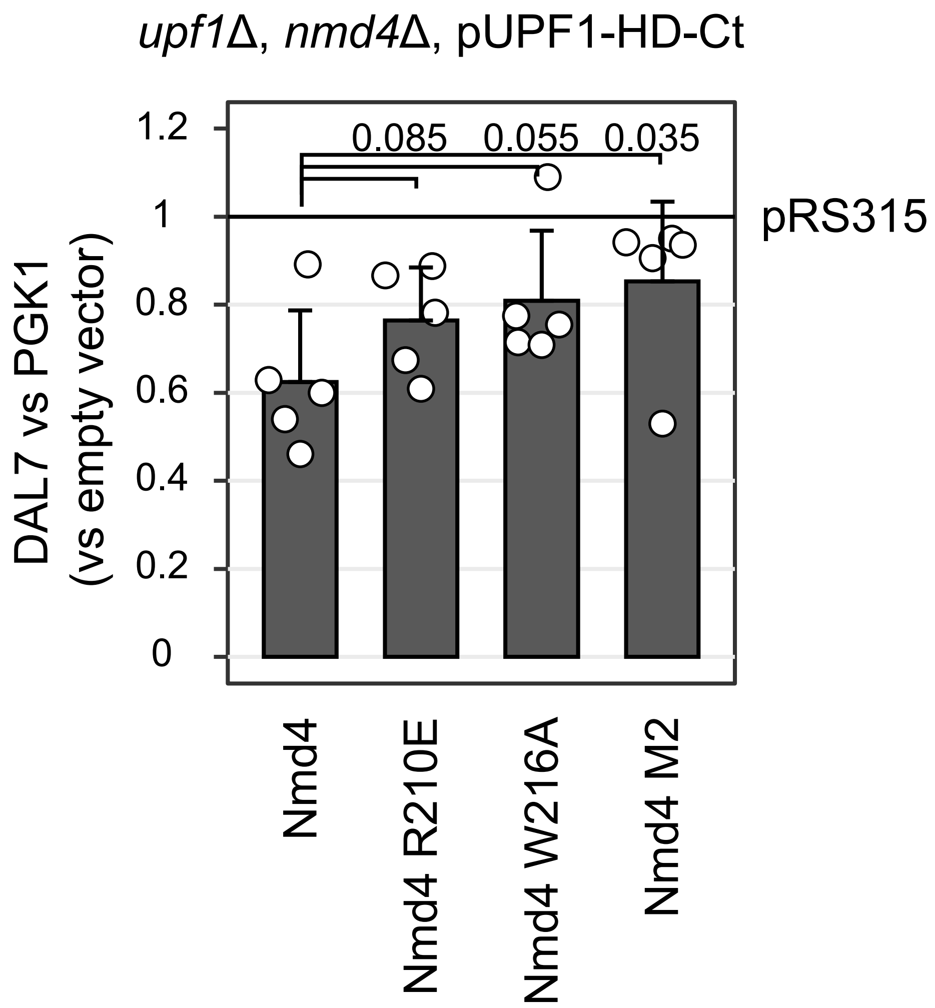

Figure legend : Nmd4 deficient for binding to Upf1 is impaired for its NMD role. A *upf1Δ/nmd4Δ* strain was transformed with a yeast CEN vector (URA3) expressing a truncated WT version of the Upf1 protein lacking the CH domain (pUPF1-HD-Ct), which can partially complement NMD, and a yeast CEN vector (LEU2) expressing Nmd4 WT, single point mutants (R210E or W216A) or the double mutant (M2) that are affected in their interaction with Upf1. One natural NMD substrate, the uORF containing DAL7 transcript was used to estimate NMD efficiency and its level was measured by RT-qPCR. The obtained values were normalized for total RNA amounts using PGK1 and all the results were adjusted to the situation in which full length Upf1 was expressed (marked "pRS315"). The experiments were repeated five times and the individual results are indicated as dots. Error bars represent standard deviation. The p-values correspond to a one-tailed t-test (using the null hypothesis that the values obtained for wt are no less than those for wt).
