# Supplementary material for: Structure of the Nmd4-Upf1 complex supports conservation of the nonsense-mediated mRNA decay pathway between yeast and humans
Source: PLoS Biol. 2024 Sep 27;22(9):e3002821. doi: 10.1371/journal.pbio.3002821 (PMC11463774; doi:10.1371/journal.pbio.3002821)
Supplement: Attachment — Submitted filename: 144614_1_rebuttal_2987751_sbm31y.docx [file pbio.3002821.s032.docx]

We would like to express our gratitude to the reviewers for their comments, which helped us to improve the quality of our manuscript. Below are the responses to each comment. We hope that these responses will satisfy the reviewers.

**Reviewer #1**

**Evidence, reproducibility and clarity**

Summary:
The nonsense-mediated mRNA decay (NMD) is and RNA quality pathway that eliminates mRNAs containing premature termination codons. Its mechanism has been studied for several decades but despite enormous progress we still don't have a satisfactory model that would explain most of the published observations. In particular, the mechanism has been proposed to differ substantially between yeast and metazoa. Yeast Nmd4 protein was previously shown to be involved in NMD, to interact with UPF1 and exhibit similarities with metazoan SMG6 and SMG5/7, that are normally believed to be specific for metazoan NMD (Dehecq et al., EMBO J, 2018). Barbarin-Bocahu et al now describe the crystal structure of the complex between the yeast UPF1 RNA helicase and Nmd4. Importantly, the authors show that interaction is required for NMD activity and increases the ATPase activity of UPF1. Barbarin-Bocahu et al equally show that this interaction and its role in NMD is conserved in the human UPF1-SMG6 complex, thus providing additional novel evidence for universal conservation of the NMD mechanism in eukaryotes.
The manuscript carefully combines biochemistry, biophysics with functional in vivo studies. In my opinion, all the experiments are very well executed, generally convincing and interpretations appear correct, so the manuscript is certainly suitable for publication.
I have included some suggestions below that I believe could strengthen the manuscript and enhance our confidence in the findings.

We are grateful for the useful suggestions that have enabled us to improve our manuscript.

*Major comments:

Page 7 - "Since the D1353A mutation completely abolishes the enzymatic activity of SMG6 (34), this strongly suggests that the PIN domain of Nmd4 is not endowed with endonucleolytic activity. " Could/was the endonucleolytic activity of NMD4 be tested?*

We agree with this important point. Our statement is based on previous site directed mutagenesis experiments on the PIN domain of human SMG6 (Galvan et al; 2006; EMBO Journal; PMID : 17053788 / Eberle et al; 2008; Nat. Struct. Mol. Biol.; PMID : 19060897), which showed that D1353 is the critical residue of SMG6 active site involved in the endonuclease enzymatic activity. Given that in yeast Nmd4 proteins, the corresponding residue is hydrophobic (Leu112 in *S. cerevisiae* Nmd4 and Phe114 in *Kluyveromyces lactis* Nmd4) and therefore cannot participate directly in catalysis, we assume that yeast Nmd4 proteins have no endonucleolytic activity.

Furthermore, despite decades of research in this field, no endonucleolytic activity has been described as being involved in the NMD pathway of *S. cerevisiae* (the model system in which the NMD mechanism was discovered in the 1970’s), whereas it has been well characterized in the NMD pathway of metazoans for more than twenty years (Gatfield and Izaurralde; Nature; 2004; PMID : 15175755 / Huntzinger et al; RNA; 2008; PMID : 18974281 / Eberle et al; Nat. Struct. Mol. Biol.; 2009; PMID : 19060897 / Lykke-Andersen et al; Genes Dev.; 2014; PMID : 25403180). Our attempts to demonstrate an endonucleolytic activity of purified Nmd4 *in vitro* were not successful. This negative result could be due to many reasons, including loss of enzymatic activity in the tested buffer, the absence of an important cofactor or the choice of the tested RNA. For these reasons, we prefer not to include this type of negative result in the current manuscript.

We hope that, on the basis of the above informations, the reviewer will agree that further substantial efforts to demonstrate a hypothetical endonucleolytic activity of Nmd4 are unlikely to be fruitful. Moreover, we believe that even if yeast Nmd4 turns out to behave as an endonuclease, this fact does not change the main message of the manuscript.

Page 10 - The two proteins bind RNA with reasonable affinity. The complex binds polyU RNA with Kd of 0.44 μM . The authors suggest, based on structure superpositions, that RNA fragments bound to the PIN domain and Upf1-HD have opposite orientations. But since they have the complex ready to crystallize, did they attempt to determine the structure with of the complex with RNA? The complex is quite small (~100 kDa with RNA) but it could be even visible by cryo-EM. I don't insist that such a structure needs to be included but it might perhaps be easy to do and would surely strengthen the story. If it is too difficult, it could at least be mentioned that it was tried?

We agree that it would be interesting to determine the crystal structure of the complex with a short RNA fragment. Unfortunately, despite extensive efforts, we could not obtain crystals of the complex in the presence of RNA. This is probably due to the large movements of the RecA2 and 1B domains relative to the RecA1 domain observed in former studies upon RNA binding to Upf1. We have mentioned that we tried to crystallize this complex in the absence or the presence of a short oligonucleotide in our revised manuscript.

As far as single-particle cryo-EM is concerned, we are aware that recent advances in this field should make it possible to determine the structure of the Nmd4-Upf1-RNA complex, but we do not yet have the necessary expertise in this technique. Despite the interesting information that such a structure could provide, we therefore consider that this would require a very significant investment and that it is beyond the scope of this manuscript.

I think it is important to demonstrate that the structure-based mutants don't significantly impact the overall structure of the proteins (e.g. glycine residues are mutated within helices). At least gel filtration profiles with gels of the WT and mutated proteins should be shown in SI.

Thank you very much for highlighting this point. We fully agree that it is important to demonstrate that the Upf1 and Nmd4 mutants used in the *in vitro* experiments (pull-down and ATPase assays) are not affected in their overall folding. As suggested by the reviewer, we have included gel filtration chromatograms for WT and mutant proteins (Figures S2A for Upf1-HD proteins and S2B for His6-ZZ-Nmd4 proteins). These chromatograms clearly show that the different mutants behave very similarly to the WT proteins during purification, demonstrating that the overall structures of the mutants are very similar to those of the wild-type proteins. We have also included the Coomassie blue stained SDS-PAGE analysis of the proteins present in the main peak to show the purity of the final proteins.

Perhaps the main finding of this manuscript is the conservation of the UPF1-Nmd4 interaction in human UPF1-SMG6. But the interaction is only demonstrated by co-IP with ectopically expressed human proteins in human cells that contain all the other human proteins as well. It would probably be more convincing to demonstrated the interaction in pull-downs with purified proteins as done for the yeast complex.

Thank you for highlighting what we consider to be one of the most interesting findings presented in our manuscript. We agree that pull-down experiments using pure protein fragments expressed in *E. coli* would have been ideal to further confirm our co-IP results and to validate that mutations do not affect the overall structure of SMG6. Unfortunately, despite considerable efforts, we were unable to express sufficient quantities of the SMG6-[207-580] fragment or shorter versions as soluble proteins in *E. coli*. Indeed, Elena Conti's laboratory had the same experience according to a statement in a paper on SMG6 (Chakrabarti et al; 2014 Nucleic Acids Research; PMID: 25013172), indicating that this region protein is very difficult to work with. As we have not yet set up protein over-expression techniques in human cells or baculovirus-infected insect cells in our laboratory, we have not been able to try these expression systems to express these SMG6 domains. These are the reasons why we decided to demonstrate this interaction by co-IP experiment using ectopically expressed tagged proteins in human cells and all appropriate controls.

In addition, using purified proteins would enable testing whether the mutations in SMG6 don't affect the overall structure of the mutants compared to the WT.

We agree that this is an important issue. Several bioinformatics tools, including AlphaFold2 (identifier: AF-Q86US8-F1), predict that the human SMG6-[207-580] fragment is largely unstructured (see panel A of figure below). Furthermore, the pLDDT values or confidence scores for this region in the AlphaFold2 model are very low (below 50), indicating that the structure of this region is poorly predicted (see panel B of figure below). Therefore, biophysical techniques to assess that the overall structure of this fragment is not affected by the introduced mutations are very limited. However, we did not observe reduced levels of SMG6 mutants compared with WT in human cells expressing these variants (Fig. 4B and S4), so we believe that these mutants behave similarly to the wild-type fragment, as is often postulated by scientists for *in cellulo* studies. Furthermore, if these mutants drastically affect the overall structure of SMG6, we would expect NMD to be strongly affected, resulting in a notable accumulation of NMD RNA substrates in our *in cellulo* experiments when the effect of the double mutant (M2) is compared to that of the SMG6 WT protein (Fig. 4C). This was not the case. On the basis of all these elements, we assume that the overall structure of the SMG6 protein is not affected by these mutations.

**Figure for reviewing purpose : Model of the three-dimensional structure of human SMG6 protein generated by AlphaFold2.**

A. Model of human SMG6 protein (green) with the region 207-580 used in our study colored in red.

B. Model of human SMG6 protein (green) colored according to the pLDDT values. Orange : pLDDT < 50; yellow : 50 < pLDDT < 70; cyan : 70 < pLDDT < 90; Blue : pLDDT > 90.

Since the detected similarity to Nmd4 is only in a region covering residues 440-470, why is the tested construct much larger (207-580) including extra, large disordered regions.

For *in cellulo* studies, it has previously been shown that the SMG6-[207-580] fragment is expressed as a stable protein in human cells and is responsible for the phospho-independent interaction between UPF1 and SMG6 (Chakrabarti et al; 2014; Nucleic Acids Research; PMID: 25013172). As our aim was not to reduce this SMG6 region to a shorter peptide but to conduct an amino acid-level analysis by site-directed mutagenesis, we decided to perform our experiments using the same SMG6 domain as Conti's laboratory and to mutate conserved residues on this fragment.

Finally, the most convincing way to show and characterize the human UPF1-SMG6 interaction would be an X-ray structure. It might be feasible to crystallize human UPF1 HD domain with a SMG6 peptide. Or at least an Alphafold model could be included? I had a quick try just with the Colabfold and using the HD domain and the SMG6 peptide, Alphafold can predict convincingly the binding of the region around W456 and in some models even around R448. I think that this would strengthen the conclusions in this part of the manuscript.

We agree that determination of the crystal structure of human UPF1 HD linked to this region of SMG6 protein interaction would have further supported our conclusions on the conservation of UPF1-Nmd4 interaction in human UPF1-SMG6. However, due to the SMG6 expression problems mentioned above, we were unable to reconstitute the human complex *in vitro*, which precluded crystallization assays.

Based on this suggestion, we generated a model of human UPF1-HD bound to the 421-480 region of human SMG6 using AlphaFold2 Colabfold. Of the various models proposed (25 in total), most are very similar and show that the side chains of R448 and W456 of SMG6 bind to regions of human UPF1 corresponding to the region of the yeast protein that interacts with R210 and W216 of Nmd4. This model is consistent with our hypothesis and we have decided to include it in the revised manuscript as suggested (Fig. EV6). We thank the reviewer for this constructive comment.

We have added the following text to mention this model : « Based on this observation, we generated a model of the complex between human UPF1-HD and the region 421-480 of SMG6 using AlphaFold2 software (1,2). In this model, the SMG6 fragment binds to the same region of UPF1-HD as the Nmd4 « arm » (Fig. EV6). In particular, the R448 and W456 side chains of SMG6 match almost perfectly with R210 and W216 side chains of *S. cerevisiae* Nmd4, suggesting that this conserved region from SMG6 is involved in the interaction between the SMG6 and UPF1-HD proteins. »

Does the SMG6 addition also increases the ATPase activity of UPF1?

This is a very good point and we agree that the results of such an experiment may have further supported our conclusions about the conservation of the Upf1-Nmd4 interaction in human UPF1-SMG6. Unfortunately, due to the SMG6 protein expression problems mentioned above, we could not perform these *in vitro* experiments.

Minor comments:
Examples of electron density omit maps of the key interaction interfaces should be shown in Supplementary Information for the reader to be able to judge the crystallography data quality.
Following this suggestion, we have added two panels showing electron density omit maps of residues at the interface in Fig. S1. We hope that this will convince the reader of the quality of our crystallographic data. We have also added the following sentence to the main text : « The overall quality of the electron density map allowed us to unambiguously identify the residues of the two proteins involved in the formation of the complex (Fig. S1A-B). »

I suggest to add the Kd values to ITC panels for clarity in main and EV figures.
We have taken this suggestion into account for figures 2A and EV5.

On page 10: What experiment is this referring to : "This is in agreement with our ITC experiments (carried out in the absence of a non-hydrolyzable ATP analog), which revealed no major synergistic effect between the two proteins for RNA binding." Results in EV4A? Or some other not shown data? The results in EV4A do show an increase in RNA binding when both proteins are in a complex.

Thank you for your comment. We realize that this sentence was not clear. We refer to the ITC data for the interaction of Upf1-HD, Nmd4 or the complex with RNA (Fig. EV5A). These data show a 2.3-fold increase in the affinity of Upf1 for RNA in the presence of Nmd4, which we consider to be a notable effect but not a major one. Based on the second reviewer’s comments that our comparison between Nob1 and the PIN domain of Nmd4 is not convincing, we have decided to delete this speculative section, which did not address an important point in our current study. We will address this point using more direct and sophisticated methods in future work.

On page 16, "organsms" should be" organisms"
Typo corrected.

In certain figure legends the panel labels (A,B,C..) are missing (e.g. Fig 3, EV1, EV5).
We apologize for this problem ,which was due to a conversion problem when preparing the PDF file of the submitted article. This problem has now been corrected.

The PIN domain structure was solved only to determine the structure of the complex? I only found it mentioned in the methods and no other mention of this structure in the main text. Maybe one sentence could be added to the results to explain why this structure was solved and how it compares to the complex structure.

We agree that we forgot to explain why we solved the structure of the PIN domain of Nmd4. The point was to help in the determination of the structure of the complex. We have added the following sentence to the main text to explain this point: « We also determined the 1.8 Å resolution crystal structure of the PIN domain of Nmd4 (residues 1 to 167) to help us determine the structure of the Nmd4/Upf1-HD complex. As this structure is virtually identical to the structure of the PIN domain of Nmd4 in the complex (rmsd of 0.5 Å over 163 C𝛼 atoms between the two structures), we will only describe the structure of this domain in the Upf1-Nmd4 complex. »

**Significance**

This is a important study, providing detailed insight into the function on Nmd4, SMG6 and UPF1 NMD. The results also point towards a conserved mechanism on NMD between yeast and human. I would like to highlight the quality of the experiments.
This study will be of great interest to people working on NMD but also more broadly to scientists working on RNA, helicases and structural biologists.

We are very grateful for the reviewer’s comments about the broad interest and overall quality of our work.

**Reviewer #2**

**Evidence, reproducibility and clarity**

In this study, the authors solved the crystal structure of the UPF1 helicase domain in complex with Nmd4. Through the structure and biochemical studies, they uncovered a region responsible for Nmd4 binding to UPF1, also important for their function in NMD. In the end, the authors also extended their findings to the human SMG6, proposing a conserved mechanism for Nmd4 and SMG6.

The mechanism of UPF1 functioning during NMD is a long-existing question. For decades, people have been trying to find out the roles of all the NMD factors during this process. This study visualized the first direct connection between UPF1 and the putative SMG6 homolog, Nmd4. Undoubtedly, it will aid our understanding of how the whole process works.

One of the limitations of this study is the conservation between Nmd4 and SMG6. Although they both have a PIN domain, Nmd4 is inactive while SMG6 is active. During NMD, SMG6 is thought to work to cut the mRNA, thus promoting the degradation of the non-functional mRNA. Therefore, Nmd4 and SMG6 may only share a similar binding mode with UPF1, however, they do not share similar functions. This study might only apply to yeast study.

We respectfully disagree with this comment. The role of SMG6 in NMD cannot be attributed solely to the endonuclease activity of the SMG6 PIN domain alone. Indeed, recruitment of the SMG6 PIN domain alone to an mRNA is not sufficient to destabilize it (Nicholson et al; 2014; Nucleic Acids Research; PMID: 25053839). This clearly indicates that other regions of SMG6 are critical for NMD. In our manuscript, we unveil the conservation of the Upf1-Nmd4 interaction in human UPF1-SMG6 (and probably more generally in metazoans) and show that this interaction plays a role in the optimal removal of NMD substrates. We strongly believe that our results are not only applicable to the study of yeast, but will fuel future studies in human cells aimed at describing the mechanistic details of the human NMD pathway.

comments: the study write in a very clear way, and most of the experiments are clear and sound. I do not have any major comments. I only have a few minor comments, listed below:
We are very grateful for the reviewer’s comments about the overall quality of our manuscript and of the experimental work.

1:The authors also solved the PIN domain of the SMG6. This is a result worth showing in the main figure.
In our study, we did not solve the structure of the human SMG6 PIN domain. This was done by Dr. Conti’s group in 2006 (Galvan et al; 2006; EMBO Journal; PMID : 17053788). This is the reason why we do not include this in the main figure. However, we have solved the crystal structure of Nmd4 PIN domain alone to help us determine the structure of the complex. Since it is very similar to the structure of the Nmd4 PIN domain in the complex with Upf1, we do not describe this structure in details. Following up the suggestion from another reviewer, we have included the following sentence mentioning that we have also determined the structure of Nmd4 PIN domain in the main text : « We also determined the 1.8 Å resolution crystal structure of the PIN domain of Nmd4 (residues 1 to 167) to help us determine the structure of the Nmd4/Upf1-HD complex. As this structure is virtually identical to the structure of the PIN domain of Nmd4 in the complex (rmsd of 0.5 Å over 163 C𝛼 atoms between the two structures), we will only describe the structure of this domain in the Upf1-Nmd4 complex. »

2：It would be easier to read if the authors could add all the binding constants directly into the ITC panels.

We have taken this suggestion into account for figures 2A and EV5.

3:I am confused with His6-ZZ. Is ZZ a protein tag?
The ZZ protein is a tag consisting of a tandem of the Z-domain from *Staphylococcus aureus* protein A. This domain binds to the Fc region of IgG and has been shown to improve expression levels and stability of recombinant proteins. In our case, it proved crucial to obtain mg amounts of the yeast Nmd4 protein and to enhance considerably its stability. We have added the following sentence in the « Materials and methods » section of the manuscript : « The ZZ-tag consists in a tandem of the Z-domain from *Staphylococcus aureus* protein A and was used as an enhancer of protein expression and stability. »

4:The comparison between Nob1 and the PIN domain of Nmd4 is not convincing for me. Since the PIN domain is not required for the binding between Nmd4 and UPF1, the conformation of the PIN domain could be a result of the crystal packing. Thus, it is still possible that Nmd4 and UPF1 bind to the same RNA. To this end, I challenge the conclusion the authors have made on the mRNA binding part.

We agree with your comment. Since this comparison is purely speculative and is not a major focus of our study, we decided to remove this section. We will address this point using more direct and sophisticated methods in future work aimed at elucidating this aspect.

5: "Showing that Nmd4 stabilizes Upf1-HD on RNA in the absence of ATP and that Upf1 is the main RNA binding factor in the Nmd4/Upf1-HD complex." As mentioned above, I don't think one can make the conclusion UPF1 is the main RNA binding factor; there shouldn't be a main and minor. Meanwhile, what will happen if you add ATP in? Or AMPPNP? Or ADP?
We agree with your comment that our current data do not allow to conclude precisely about the role of Upf1 as major RNA binding factor. We have replaced this sentence by the following one : « Whether this increase in affinity is due to a synergistic effect between both proteins or to an allosteric stimulation of one partner on the RNA binding property of the second partner remains to be clarified. ».

Regarding the role of the nucleotides on RNA binding properties of the Upf1 helicase domain or the complex, we faced precipitation problems when mixing high concentrations Upf1 and nucleotides for ITC experiments, making difficult to determine Kd values for the interaction between Upf1 and RNA in the presence of nucleotides. However, in a previous study (Dehecq et al; 2018; EMBO J; PMID : 30275269), we observed that AMPPNP did not affect the amount of Nmd4 and Upf1-HD co-precipitated by an RNA oligonucleotide, indicating that nucleotide does not significantly affect the interaction of the complex with RNA.

6: "But also that a physical interaction between Upf1-HD and the PIN domain exists in vitro, although we were unable to detect it using our various interaction assays." This also confused me, since one cannot detect the interaction in any assay, how could you be so confident there is a physical interaction? Have you tested assays which are good for weak binding?

We understand that this sentence may be confusing. The tests we have used to determine whether there is a physical interaction between the PIN domain of Nmd4 and Upf1-HD are ITC and pull-down. These are excellent methods for detecting stable interactions with dissociation constants (Kd) in the nanomolar to tens of micromolar range. These two methods did not indicate any direct interaction between the PIN domain of Nmd4 and Upf1-HD. However, we observed that the PIN domain of Nmd4 stimulates the ATPase activity of Upf1-HD to the same extent as the «  arm » of Nmd4. This is an indirect indication that the Nmd4 PIN may interact with Upf1-HD, otherwise a stimulatory effect would not be expected. Our radioactivity-based ATPase assay is very sensitive, allowing the detection of a stimulatory effect due to a transient interaction between the PIN domain of Nmd4 and Upf1-HD, which, as indicated above, could not be detected with the interaction assays used. We would also like to point out that in our ATPase conditions, Upf1-HD (0.156 µM) is incubated with a 20-fold molar excess (3.12 µM) of its partners (Nmd4-FL, Nmd4 « arm » or Nmd4 PIN). Such an excess cannot be used in our interaction tests. This could explain the stimulatory effect detected for the PIN domain of Nmd4 in our ATPase assay.

We have clarified this section by adding the following sentences: « We were unable to detect such an interaction using our different interaction assays (pull-down and ITC), which are optimal for studying interactions with dissociation constants (Kd) in the nanoM to tens of microM range. We therefore assume that a transient low-affinity interaction (high Kd value not detected by our binding assays) exists between Upf1-HD and PIN Nmd4 and can only be detected by highly sensitive assays such as our radioactivity-based ATPase assay, which was performed with a 20-fold molar excess of PIN Nmd4 domain over Upf1-HD. »

7: Figure 4B should be done in the context of the full length of SMG6 and UPF1.

**Referees cross-commenting**

*This session contains comments from both Rev1 and Rev2*

Rev1:

There seems to be a contradiction in comments on Figure 4B. I agree with Reviewer 2 that using FL proteins will be informative to see whether the FL proteins indeed interact (or not in the case of the mutants).

If one wants to use this experiment to map the interacting regions, then I think that the UPF1 HD domain and the short conserved region of SMG6 should be used. The long fragment SMG6 207-580 is not ideal for either. The short constructs would be more suited for a pull-down experiments (like done for the yeast proteins).

Rev2

Response to reviewer #1, It is necessary to use the full-length protein (FL protein) to map the interface unless they have pre-existing information to support mapping down to short fragments.

In addition, performing further structural work would be beyond the scope of this study. Given the additional time and effort required, I do not recommend doing so for this study.

Rev1:

As I said, I agree with using the FL proteins. The pre-existing information supporting the mapping comes from sequence alignments with the yeast structure and the mutagenesis. This is further confirmed by Alphafold modeling which in my opinion should be included. As I mentioned in my review, I don't insist on further structural work

Thank you very much for this comment and the discussions between reviewers, which show that we didn't explain our experimental strategy clearly. Human UPF1 has been shown to interact with SMG6 in both phospho-dependent and phospho-independent modes. In our manuscript, we focus on characterizing the phospho-independent interaction. For this reason, we cannot perform this experiment using the full-length version of SMG6 and UPF1, otherwise the effects of our point mutants on the UPF1-SMG6 interaction could be masked by the phospho-dependent interaction occurring between domain 14.3.3 of SMG6 and the C-terminus of Upf1. To circumvent this problem, we were inspired by former *in cellulo* studies, which have shown that the SMG6-[207-580] fragment is expressed as a stable protein in human cells and is responsible for the phospho-independent interaction between UPF1 and SMG6 (Chakrabarti et al; 2014; Nucleic Acids Research; PMID: 25013172). Similarly, the helicase domain of UPF1 was found to be sufficient for this phospho-independent interaction with human SMG6 (Nicholson et al; 2014; Nucleic Acids Research; PMID: 25053839). These are the reasons why we decided to use this protein domains in our *in cellulo* studies to test the effect of our point mutants on the interaction. As indicated above in an answer to one comment to reviewer #1, as our aim was not to reduce this SMG6 region to a shorter peptide but to conduct an amino acid-level analysis by site-directed mutagenesis, this is also why we decided to perform our experiments using the same SMG6 domain as Conti's laboratory and to mutate conserved residues on this fragment. We have also included the AlphaFold2 model of the complex between human UPF1 and SMG6 in our revised version.

To clarify this point, we have amended the relevant section as follows: « To determine whether this motif might be involved in the interaction between SMG6 and UPF1-HD proteins, we ectopically expressed the region comprising residues 207-580 of human SMG6 fused to a C-terminal HA tag (SMG6-[207-580]-HA) and human UPF1-HD (residues 295-921 fused to a C-terminal Flag tag; UPF1-HD-Flag) in human HEK293T cells, as these regions have previously been shown to be responsible for the phosphorylation-independent interaction between these two proteins. Compared to the full-length UPF1 and SMG6 proteins, these constructs also preclude our findings of any interference from the phosphorylation-dependent interaction occurring between the C-terminus of UPF1 and the 14-3-3 domain of SMG6. »

8: "The NMD mechanism not only targets mRNAs but also small nucleolar RNAs (snoRNAs) and long noncoding RNAs (lncRNAs) harboring bona fide stop codons but in a specific context such as short upstream open reading frame (uORF), long 3'-UTRs, low translational efficiency or exon-exon junction located downstream of a stop codon." "First, for mRNAs with long 3'-UTRs, the 3'-faux UTR model posits that a long 3 spatial distance between a stop codon and the mRNA poly(A) tail destabilizes NMD substrates by preventing the interaction between the eRF1-eRF3 translation termination complex bound to the A- site of a ribosome recognizing a stop codon and the poly(A)-binding protein (Pab1 or PABP in S. cerevisiae and human, respectively)." These are difficult to read.

Thank you for this suggestion to improve the clarity of our manuscript. We have tried to make these sentences easier to read as follow:

« The NMD mechanism also targets mRNAs, small nucleolar RNAs (snoRNAs) and long noncoding RNAs (lncRNAs) carrying normal stop codons located in a specific context (short upstream open reading frame or uORF, long 3’-UTRs, low translational efficiency or exon-exon junction located downstream of a stop codon (3-11)). »

« The first model, the 3’-*faux* UTR model posits that for mRNAs with long 3’-UTRs, a long spatial distance between a stop codon and the mRNA poly(A) tail destabilizes NMD substrates. Indeed, it would prevent the physical interaction between the eRF1-eRF3 translation termination complex recognizing a stop codon in the A-site of the ribosome and the poly(A)-binding protein (Pab1 or PABP in *S. cerevisiae* and human, respectively) bound to the 3’ poly(A) tail (12-14). »

9: please add the Ramachandran plot values.

Thank you for pointing out this omission. These values have been included in Table EV1.

**Significance**

NMD is one of the major topics in the field of gene translational regulation research. this study will be of interest to a broad audience. i am an expert in the structure study in translation. However, I have limited experience in the in vivo study of NMD substrates.

We are very grateful for the reviewer’s comments about the broad interest and the overall quality of our work.
